# Supplementary material for: Neural Isoforms of Agrin Are Generated by Reduced PTBP1−RNA Interaction Network Spanning the Neuron−Specific Splicing Regions in AGRN
Source: Int J Mol Sci. 2023 Apr 18;24(8):7420. doi: 10.3390/ijms24087420 (PMC10139058; doi:10.3390/ijms24087420)

Original gel images

Figure 1B

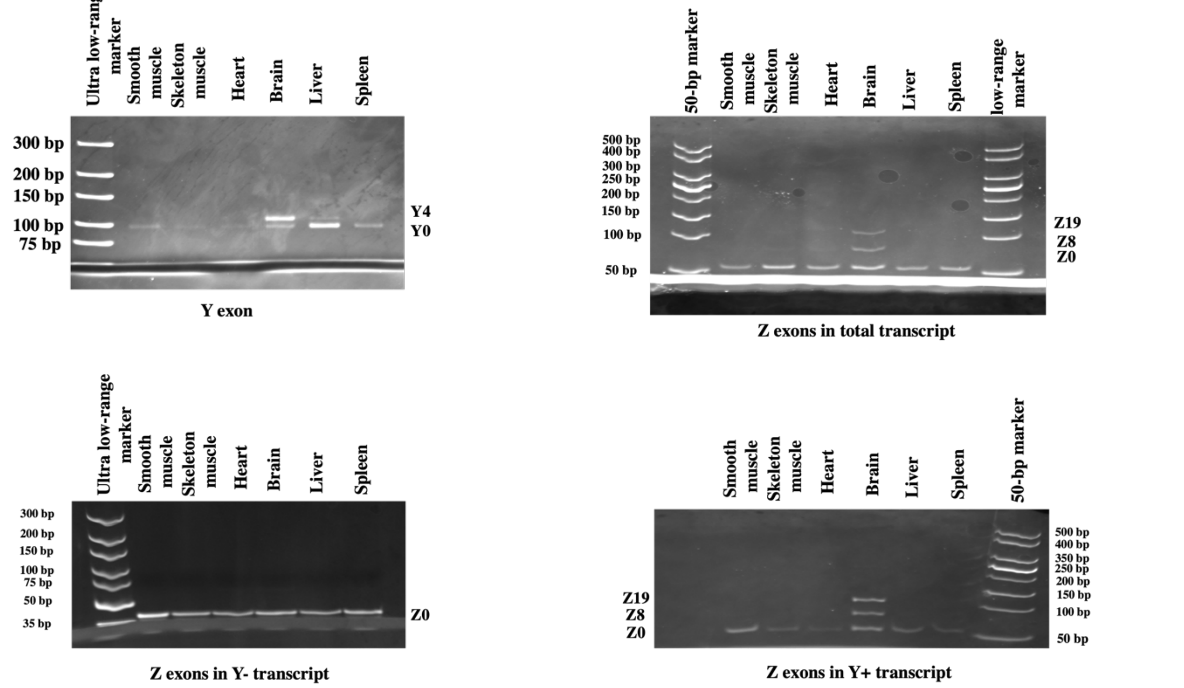

Figure 1C

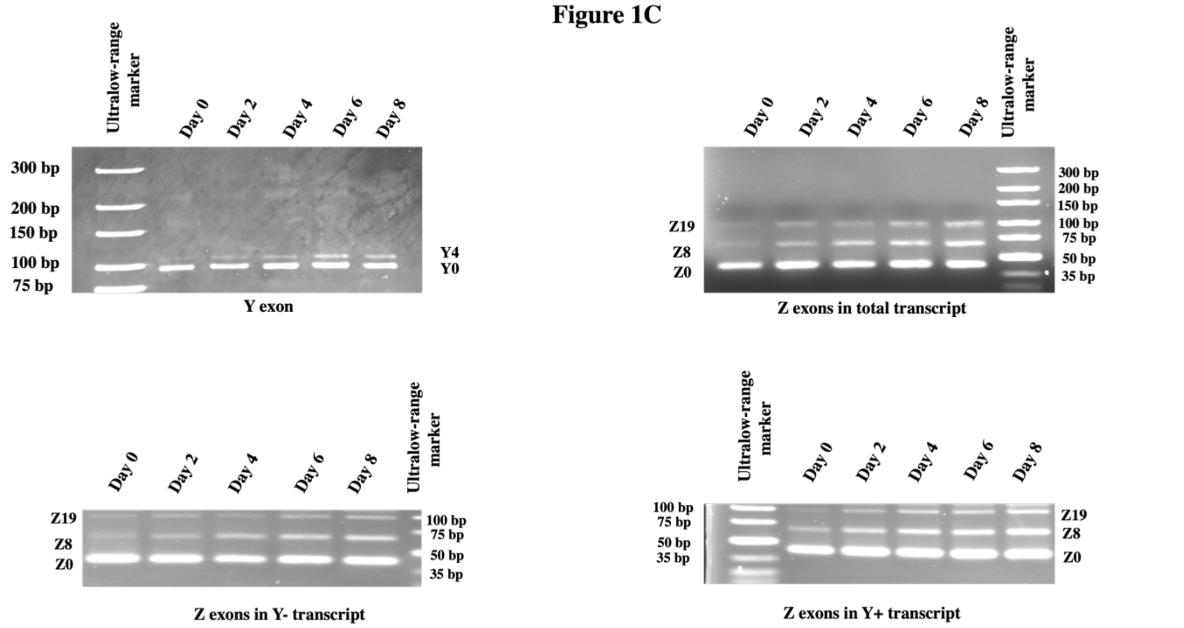

Figure 2B

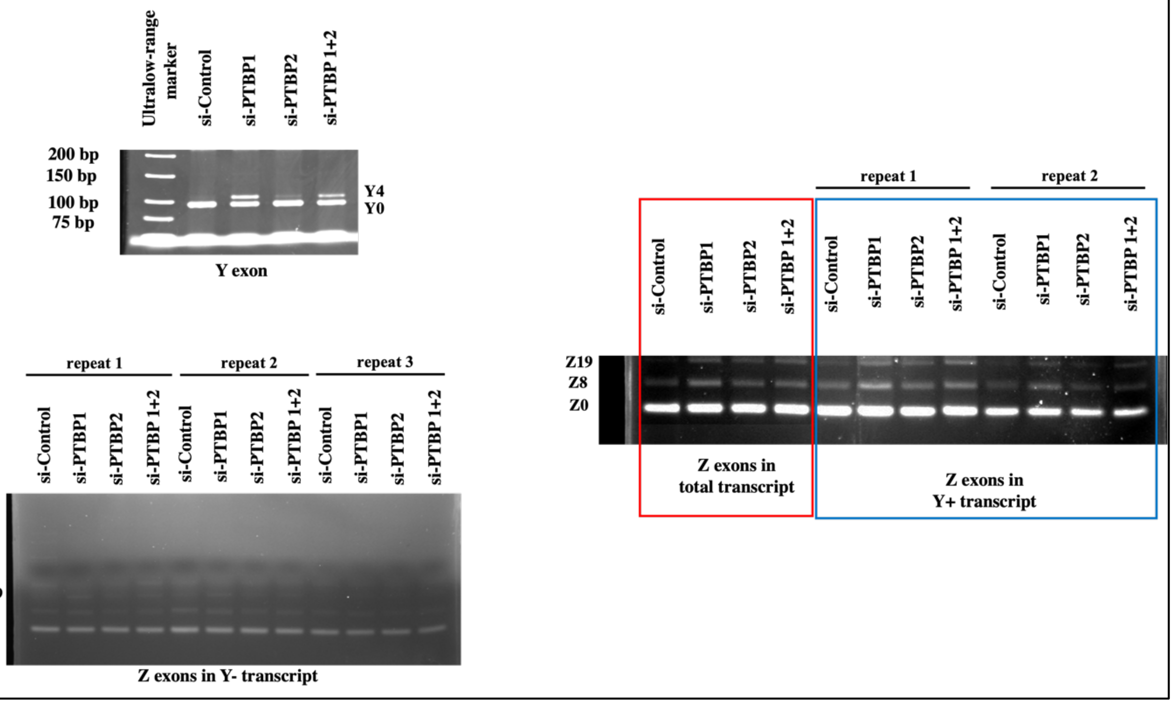

Figure 3A

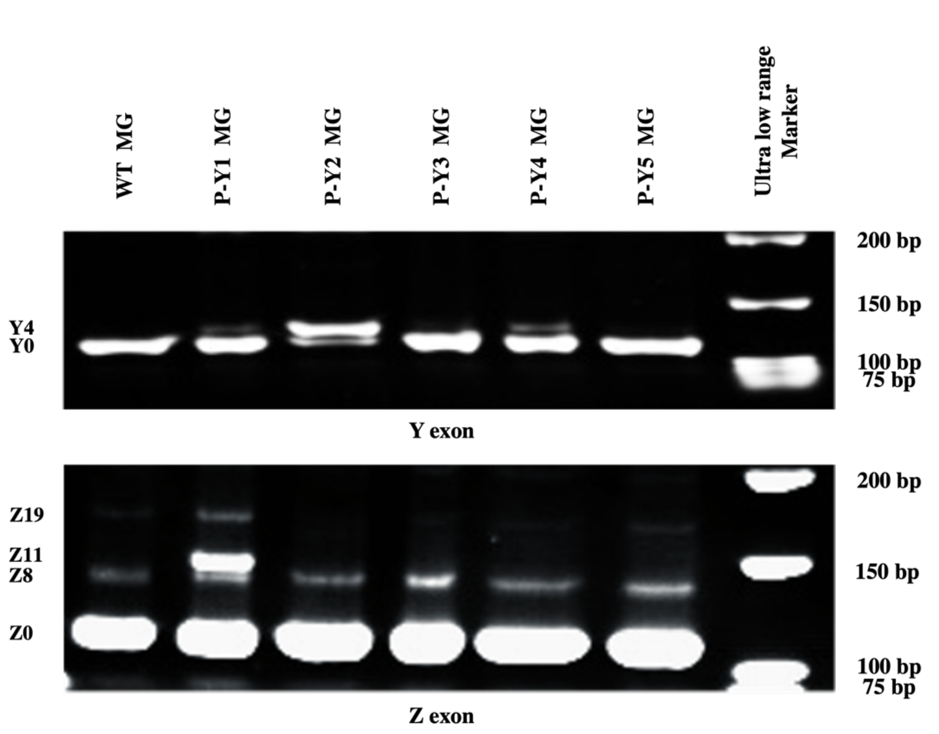

Figure 3D

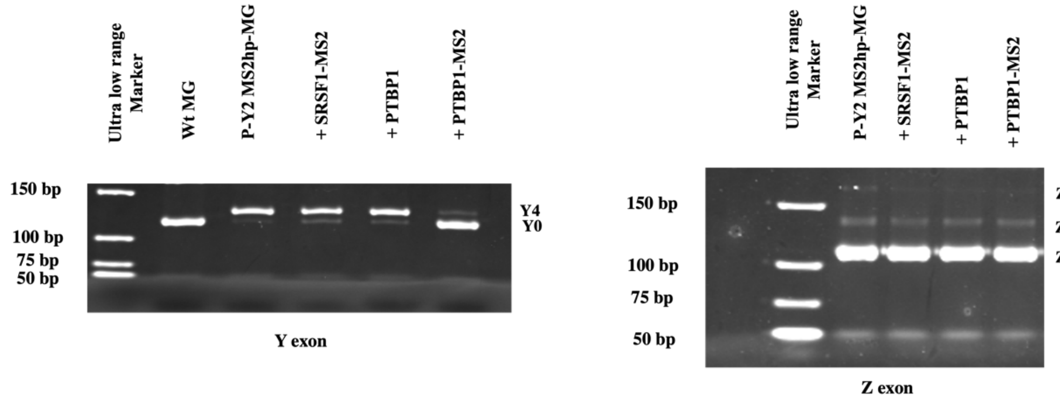

Figure 3G

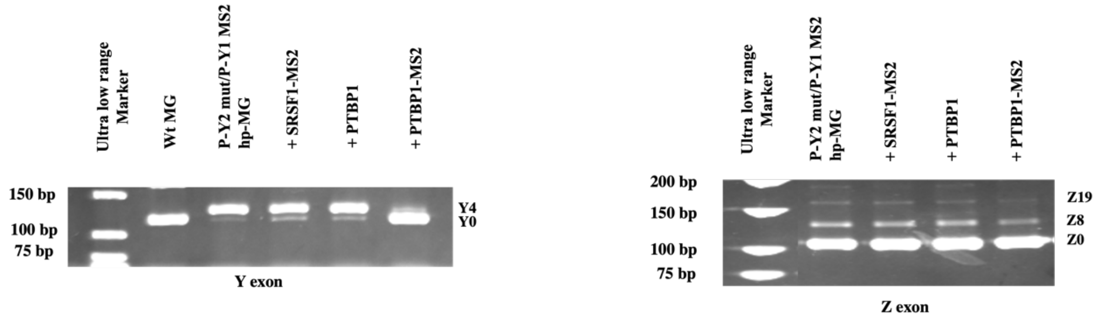

Figure 4A

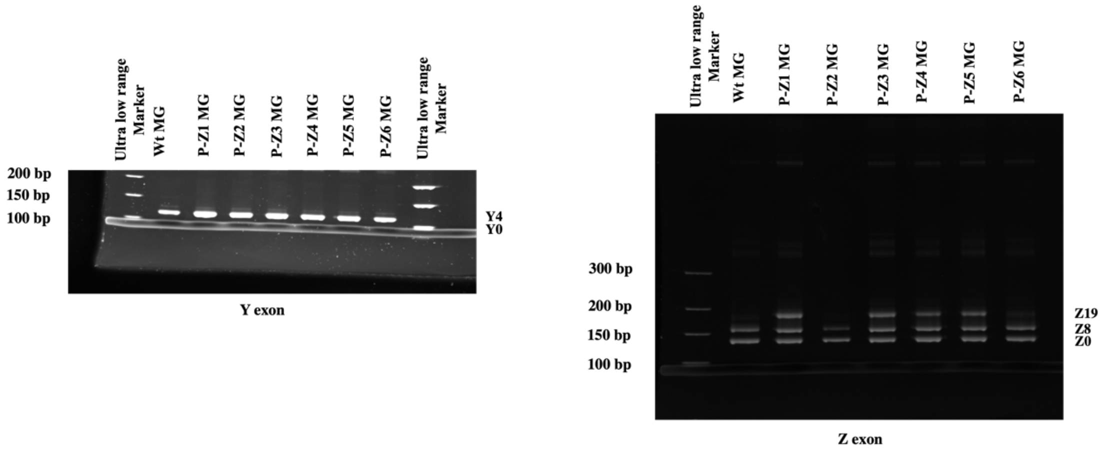

Figure 4D

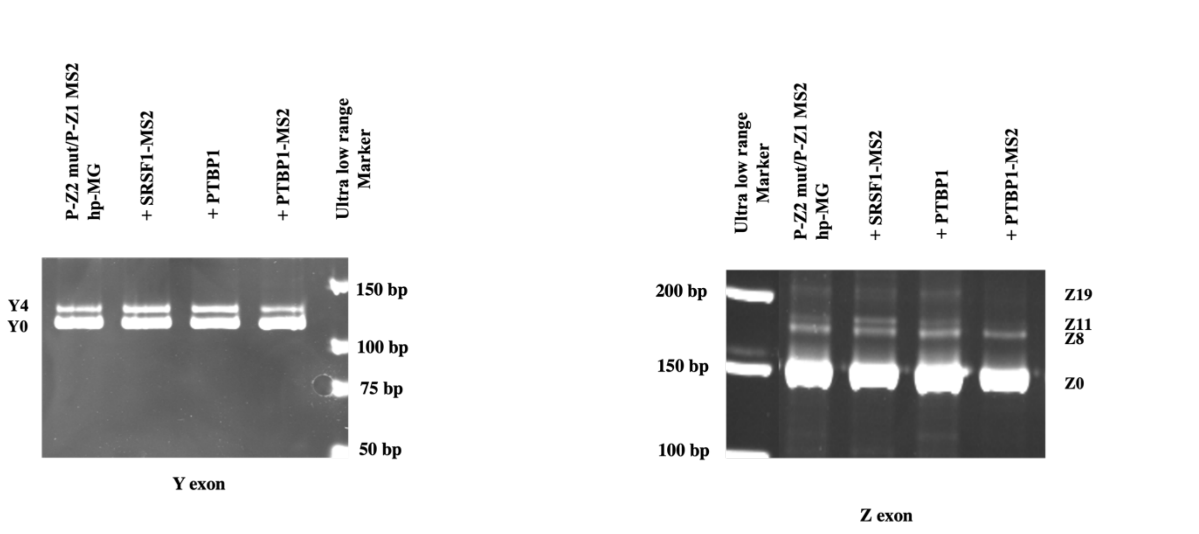

Figure 4G

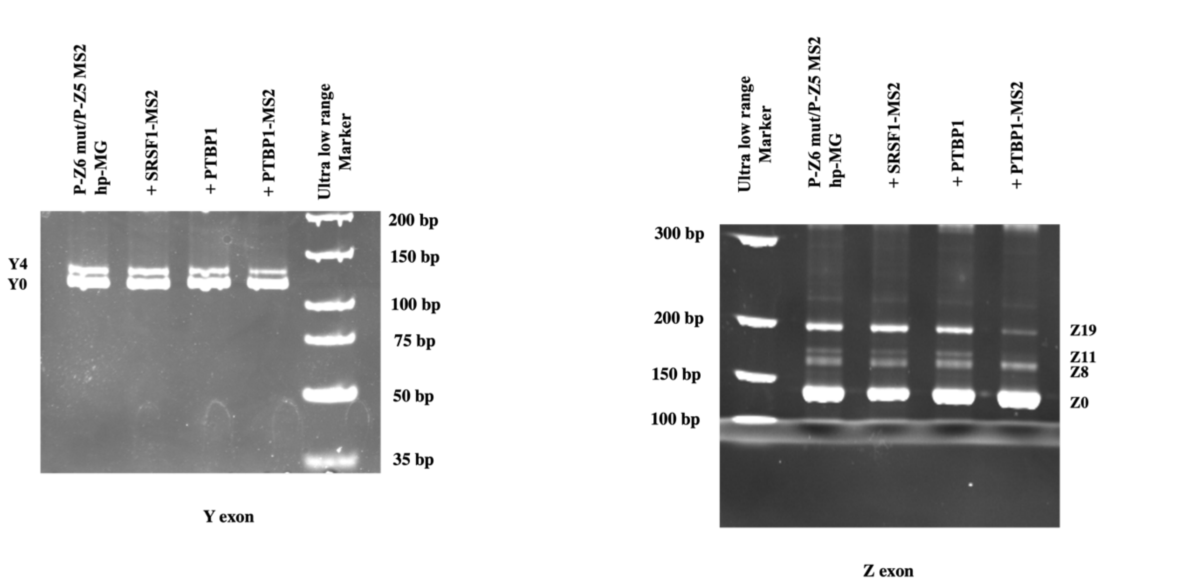

Figure 5B

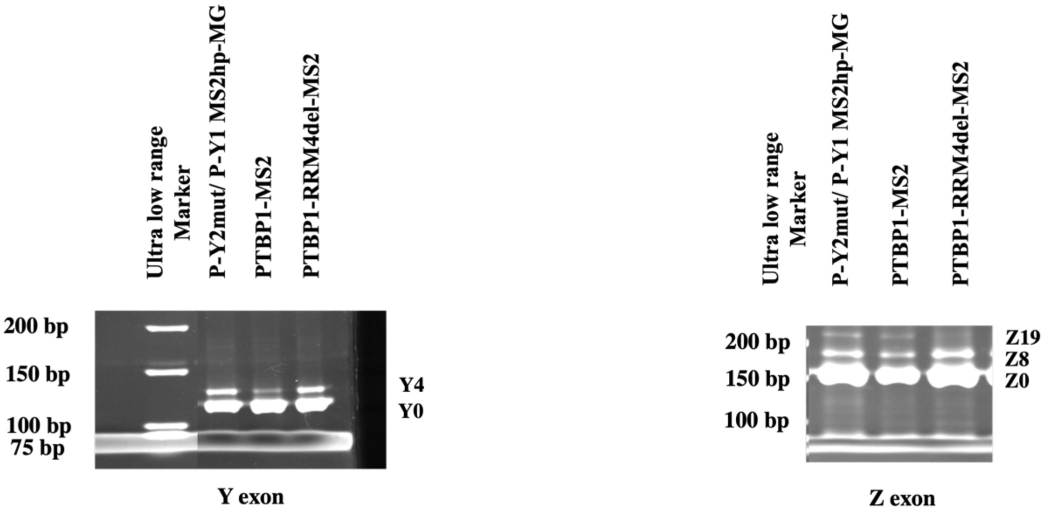

Figure 5E

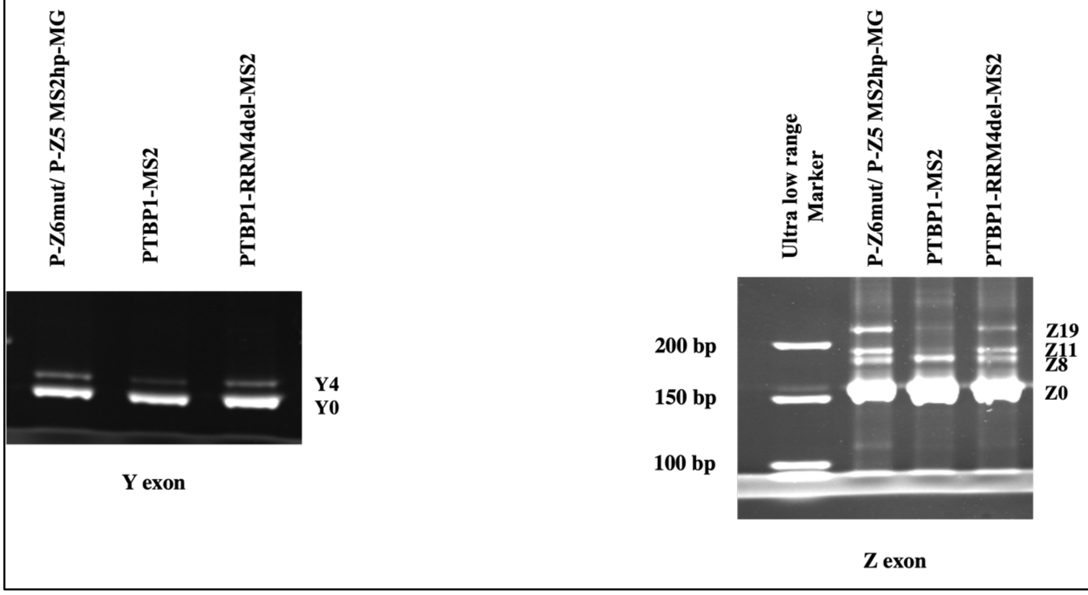

Supplementary Figure S4B

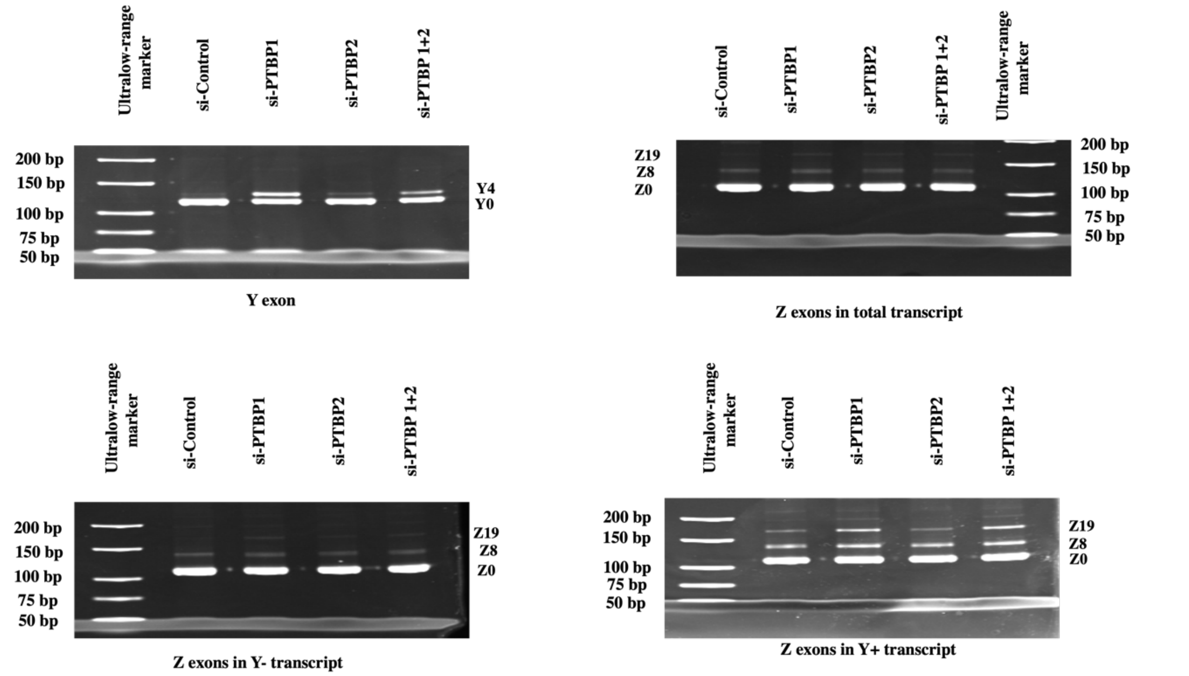

Supplementary Figure S5B

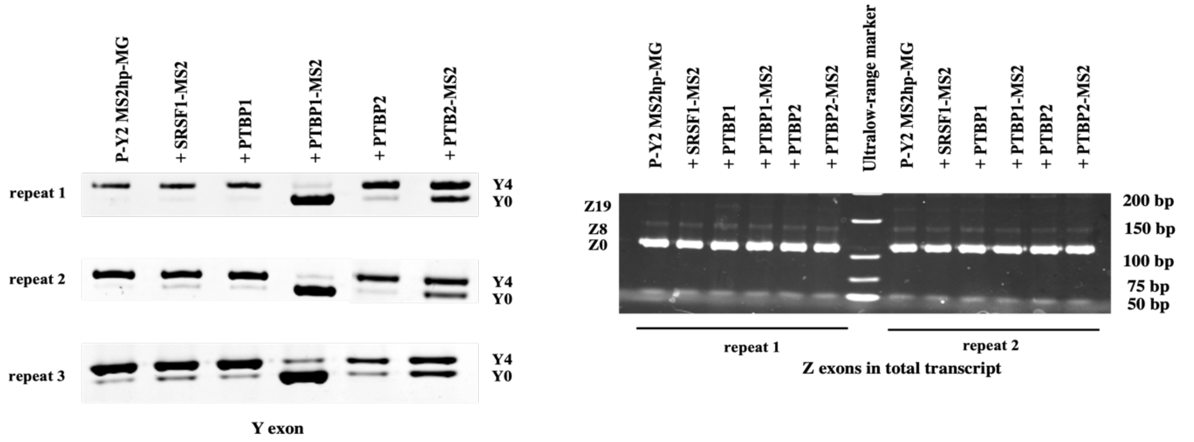

Supplementary Figure S5E

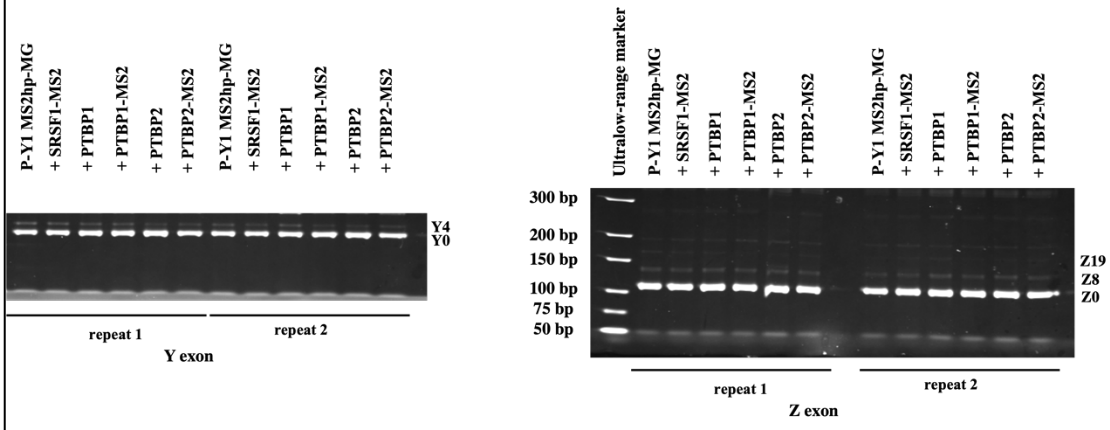

Supplementary Figure S5H

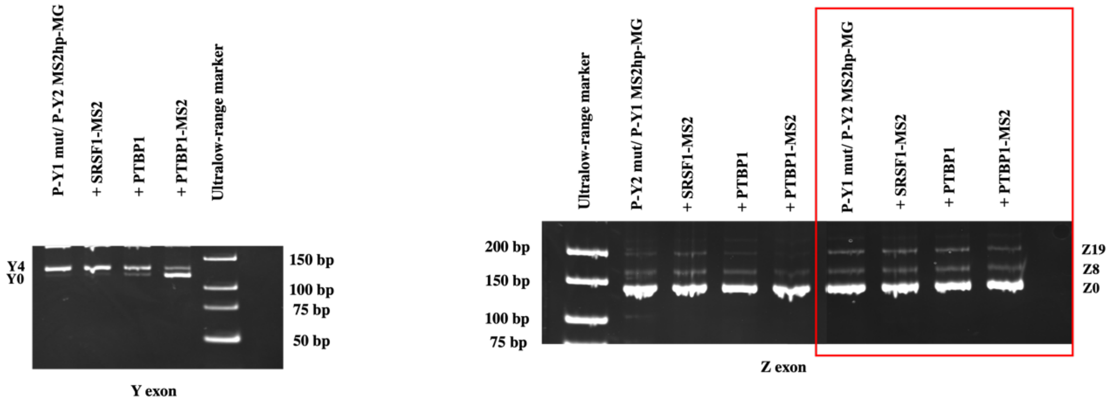

Supplementary Figure S6C

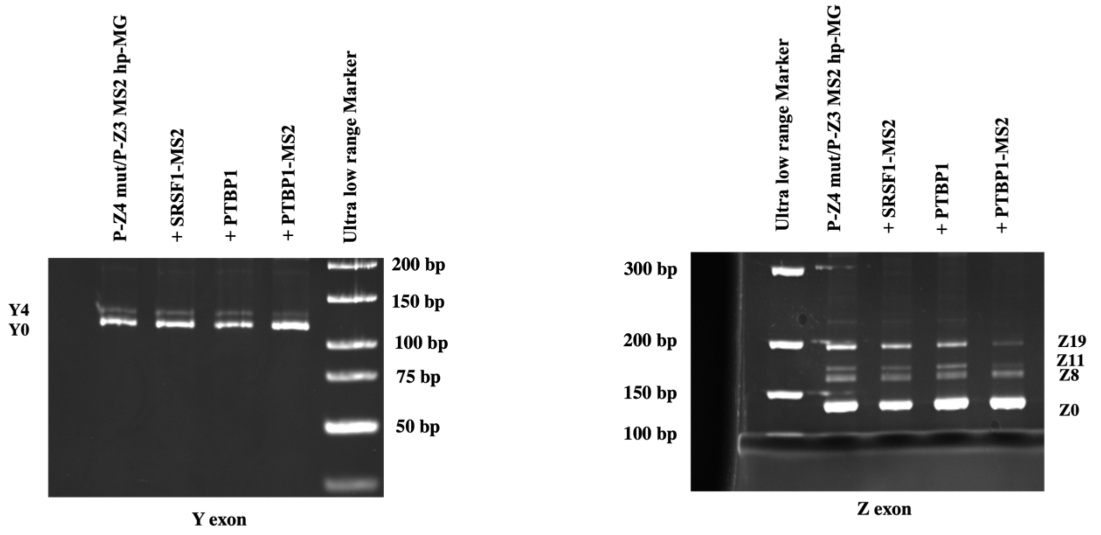

Supplementary Figure S7A

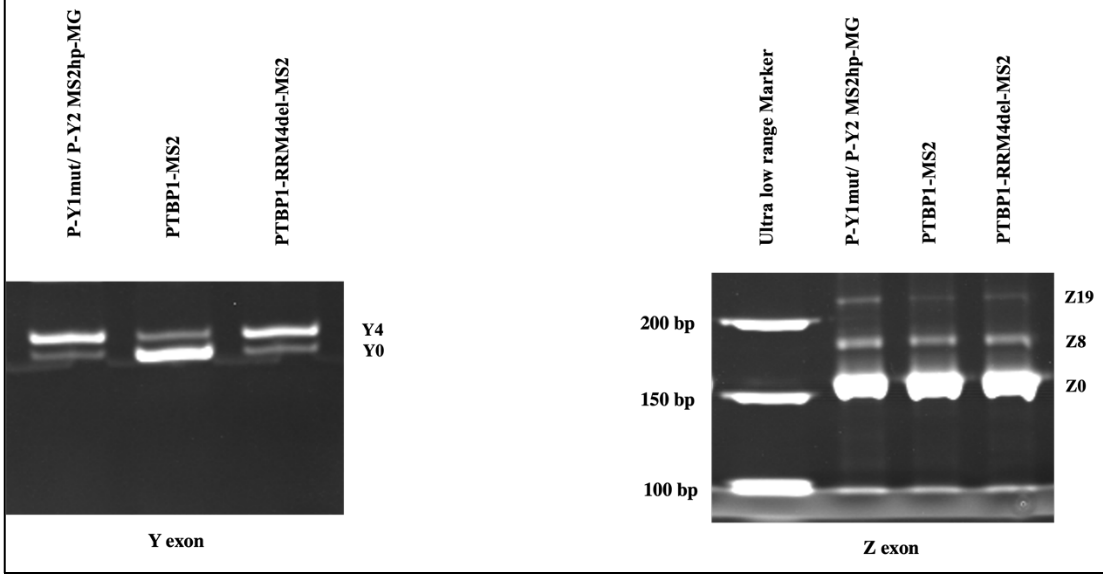

Supplementary Figure S7D

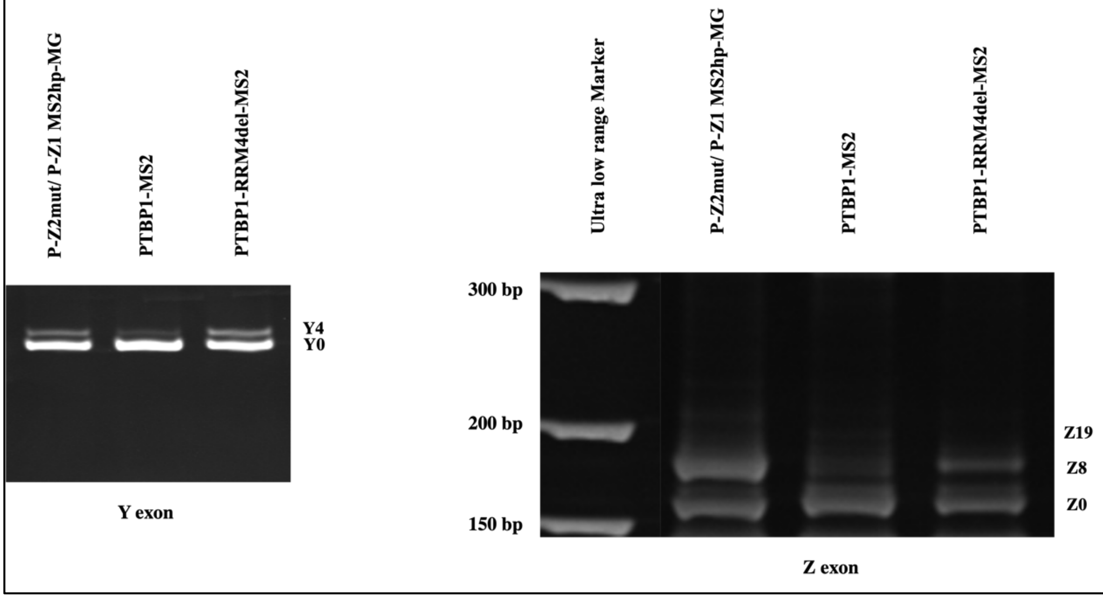

Supplementary Figure S7G

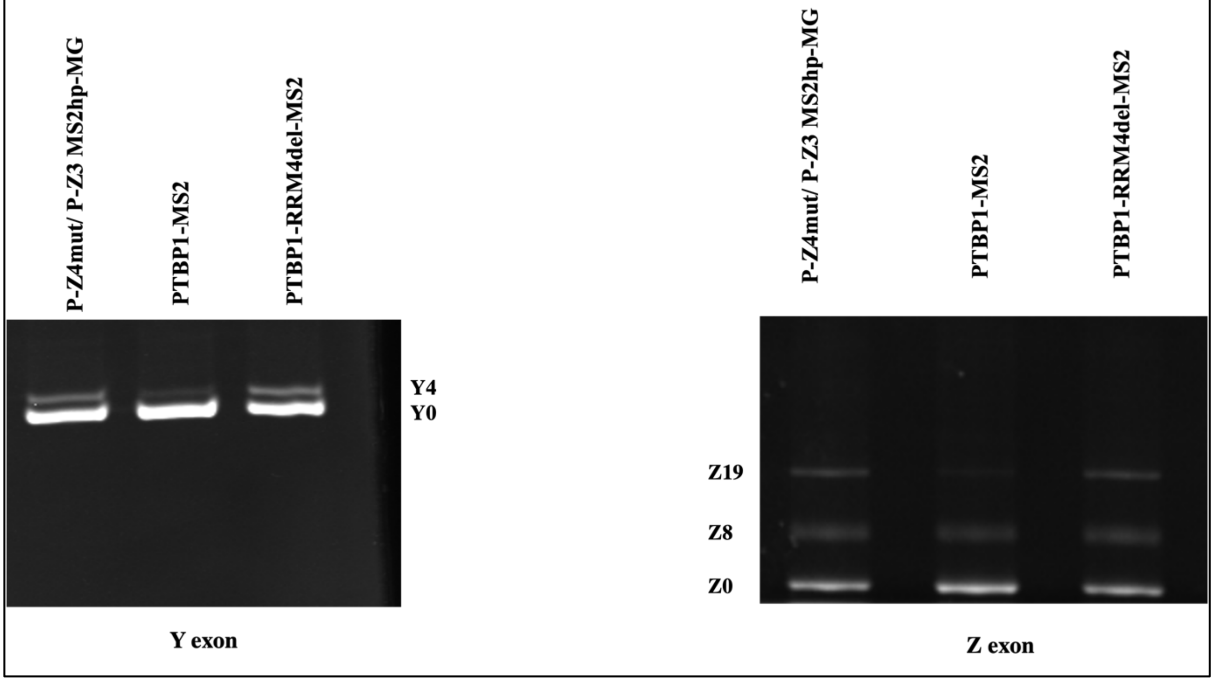

Original blots

Figure 2B: siRNA knockdown/ PTBP1 and GAPDH

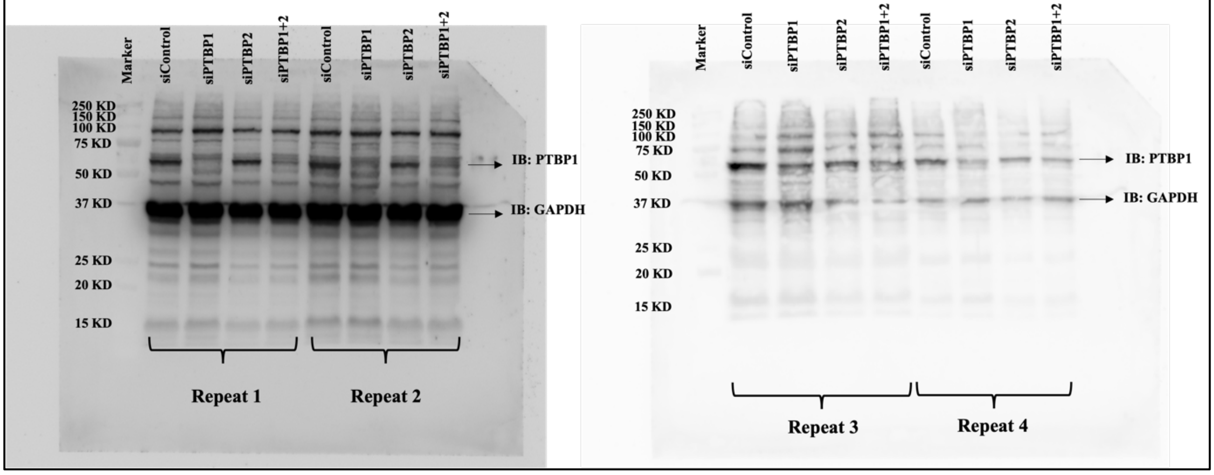

Figure 2B: siRNA knockdown/ PTBP2

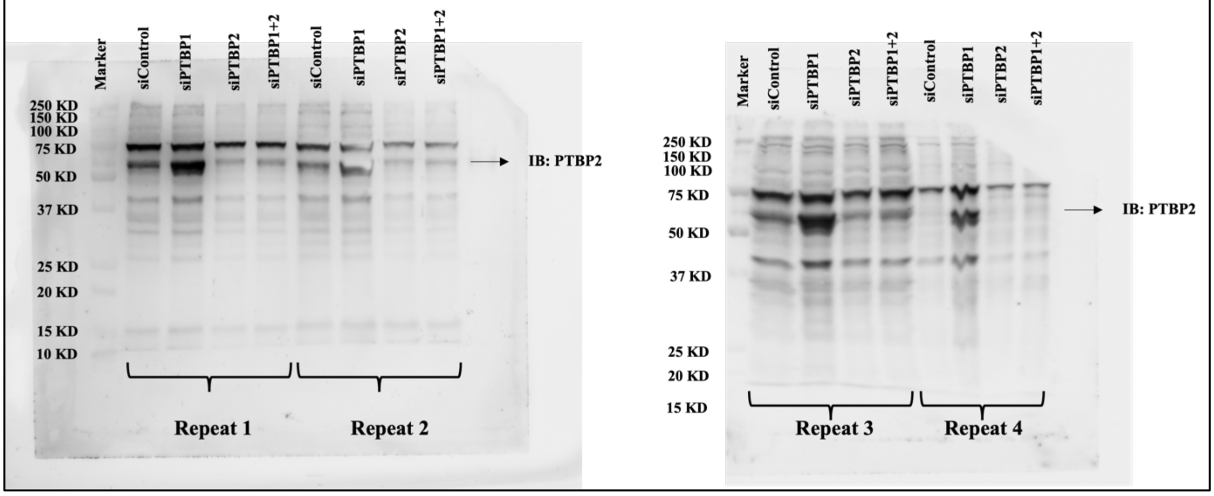

Supplementary Figure S2: neuronal cell (SH-SY5Y) differentiation/ PTBP1 and GAPDH

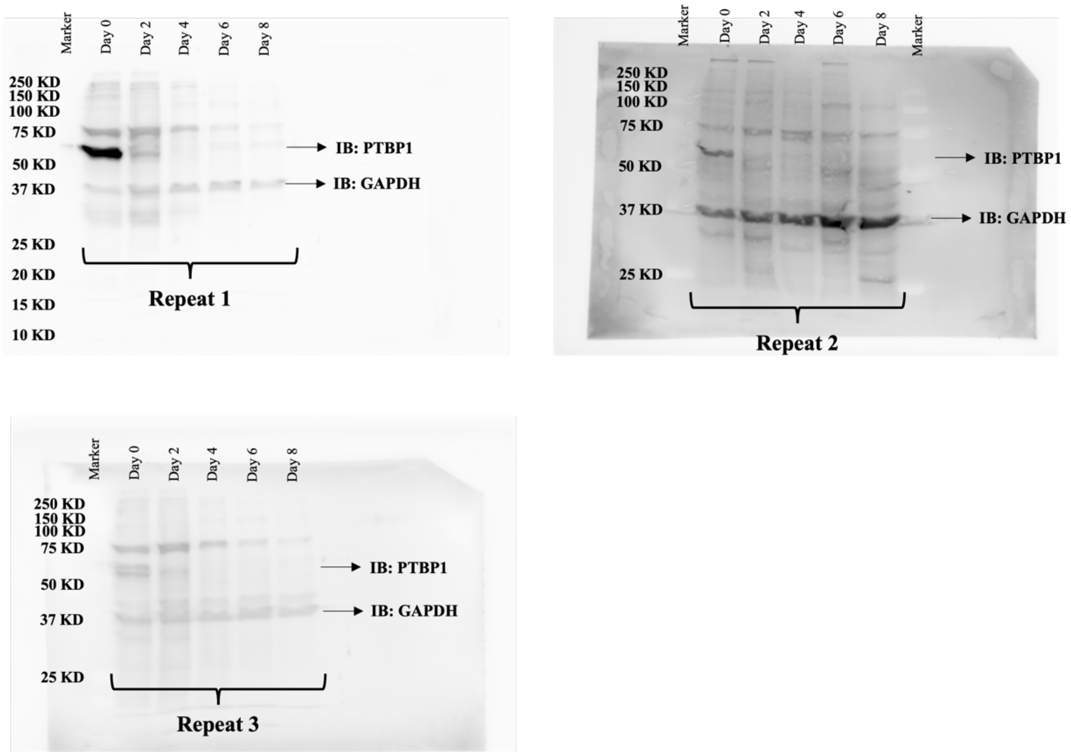

Supplementary Figure S2: neuronal cell (SH-SY5Y) differentiation/ PTBP2

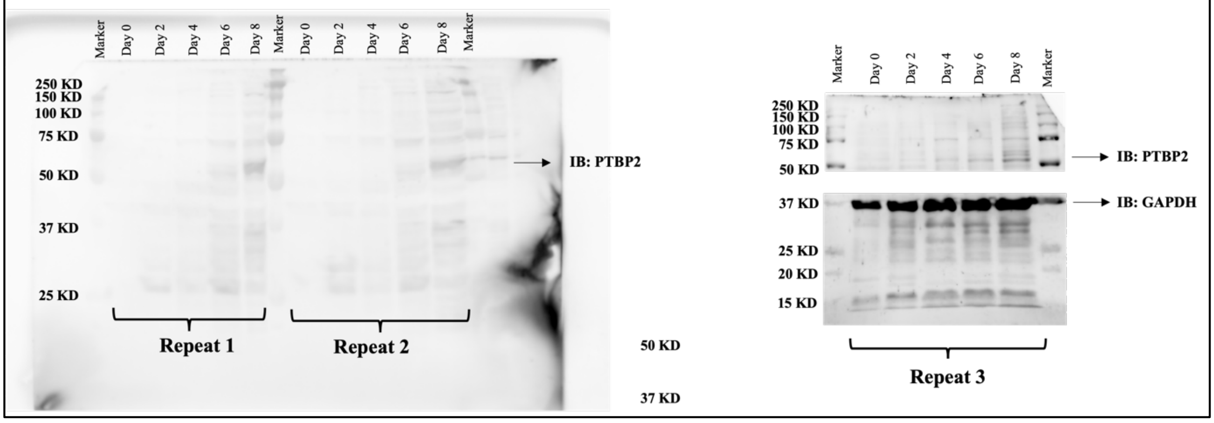

Supplementary Figure S8B

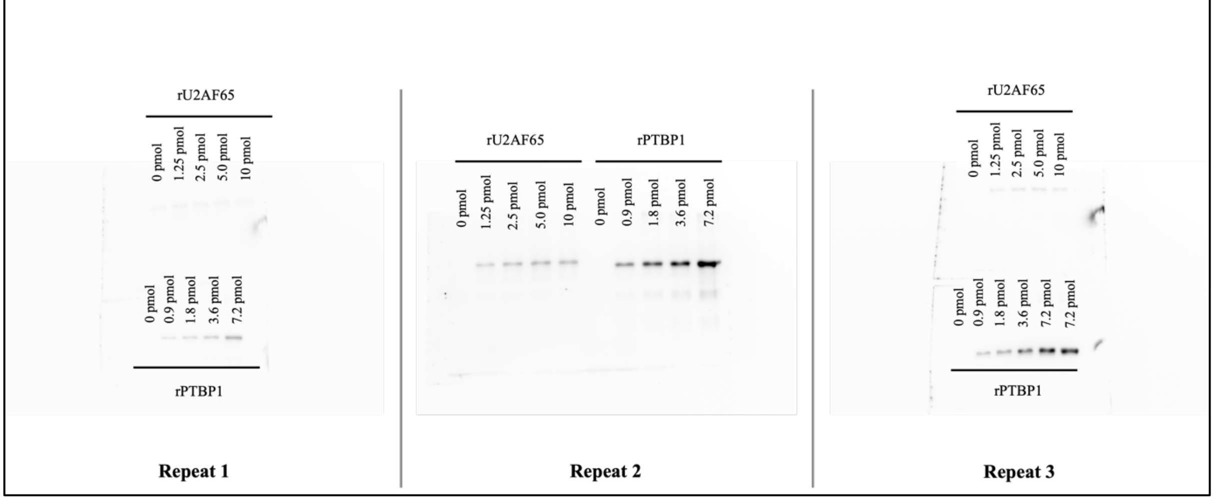

Supplementary Figure S8C

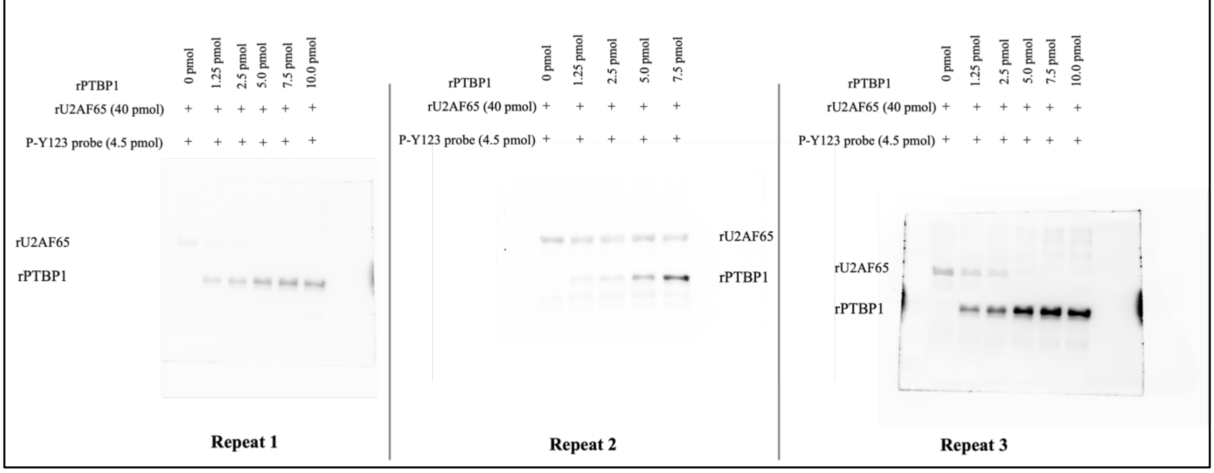

Supplement: Supplementary file 1 [file ijms-24-07420-s001.zip › ijms-2295770-supplementary/Supplementary files/Original gel and blot images.pdf]
