# Supplementary material for: Neural Isoforms of Agrin Are Generated by Reduced PTBP1−RNA Interaction Network Spanning the Neuron−Specific Splicing Regions in AGRN
Source: Int J Mol Sci. 2023 Apr 18;24(8):7420. doi: 10.3390/ijms24087420 (PMC10139058; doi:10.3390/ijms24087420)

Supplementary Figure S2

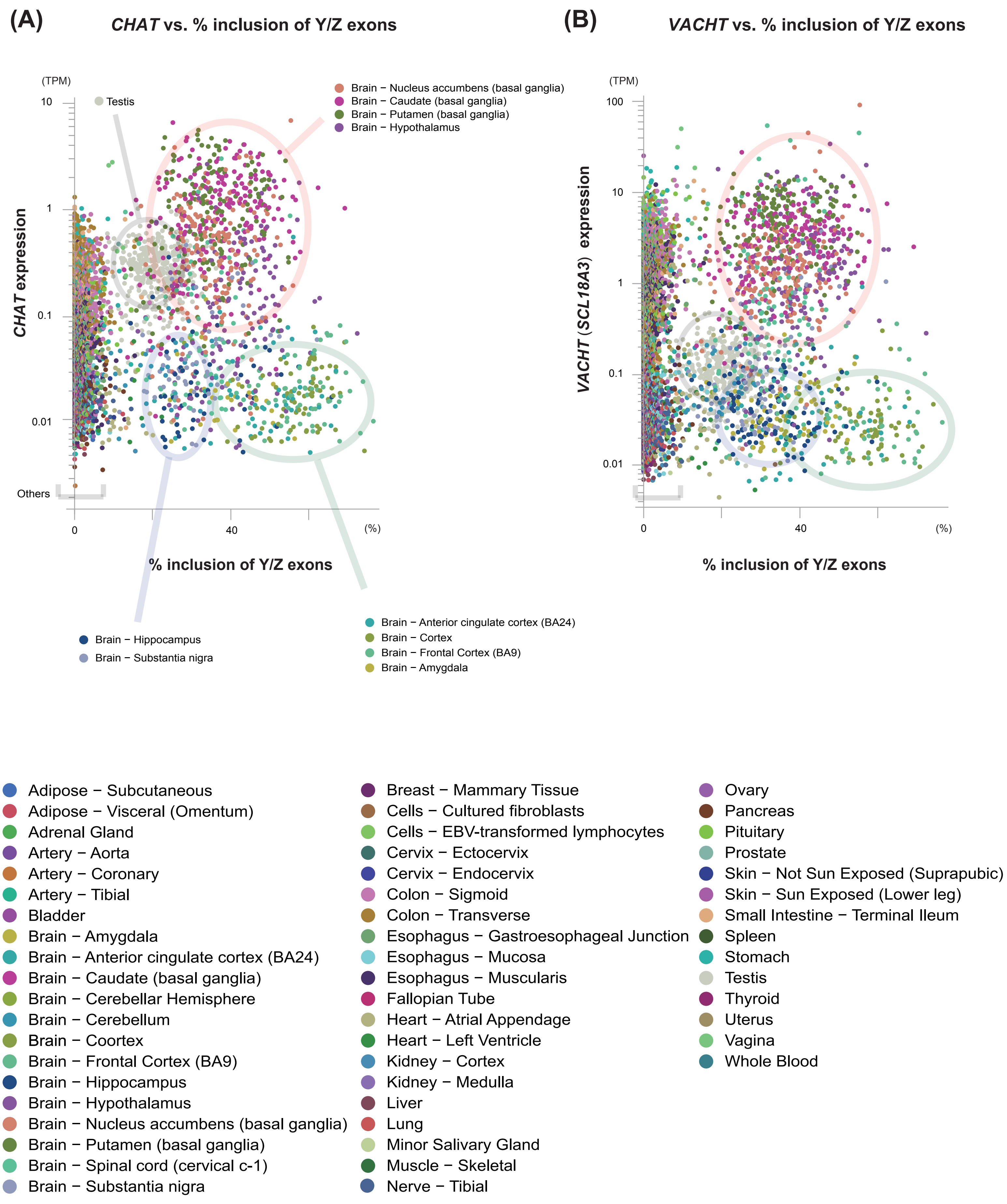

Supplement: Supplementary file 1 [file ijms-24-07420-s001.zip › ijms-2295770-supplementary/Supplementary files/Suppementary figures pdf/Supplementary figure 2.pdf]
