# Supplementary material for: Neural Isoforms of Agrin Are Generated by Reduced PTBP1−RNA Interaction Network Spanning the Neuron−Specific Splicing Regions in AGRN
Source: Int J Mol Sci. 2023 Apr 18;24(8):7420. doi: 10.3390/ijms24087420 (PMC10139058; doi:10.3390/ijms24087420)
Supplement: Supplementary file 1 [file ijms-24-07420-s001.zip › Supplementary files/ijms-2295770_Supplementary_informtations.docx]

Supplementary Information

**Supplementary Tables**

**Supplementary Table S1. Primer sequences**

| **Primers** | **5’−3’ sequences** |
| --- | --- |
| **Primers for making constructs** | |
| **Primers for making *AGRN* minigene** | |
| EcoRV−KZ−ATG.MG E30/F | GTGGAATTCTGCAGATCACCATGGTCTCACTGGAGCGAAACGG |
| EcoRV−STOP.MG E37/R | CGCCACTGTGCTGGATTTACTTGCCACTCCAGAGCACCA |
| **Primers to amplify cDNA for MS2 coat protein** | |
| KpnI−MS2/F | CTTCGGGGTACCAATGGCTTCTAACTTTACTCAGTTCG |
| BamHI−MS2/R | GTTCGCGGATCCGTAGATGCCGGAGTTTGCTG |
| **Primers for RT−PCR** | |
| **Splicing analysis of human *AGRN*** | |
| Exon Y4/F (endogenous) | TCTCACTGGAGCGAAACGGC |
| Exon Y4/F (minigene) | TGGAATTCTGCAGATCACCATG |
| Exon Y4/R (Endo/MG) | TTGAGGACGGTGTGCGGAA |
| Y− transcript/ F (Endo/MG) | GTGTGTTGGGGGAGTCCCCGGT |
| Y+ transcript/F (Endo/MG) | GAGTCCCCGAAATCCCGCAAGGT |
| Exon Z/F 1 (Endo/MG) | CTCAACGCTGTGACCGAGAG |
| Exon Z/F 2 (Endo/MG) | GCGGACCTTTGTCGAGTACCTC |
| Exon Z/R (endogenous) | TTGCTCTGCAGTGCCTTCTC |
| Exon Z/R 1 (minigene) | TTAAACGGGCCCTCTAGACTCG |
| Exon Z/R 2 (minigene) | ACCCGCTGATCAGCCTCGAC |
| BGH rev (minigene) | AACAACAGATGGCTGGCAAC |
| **Primers for marker genes** | |
| hGAPDH/F | CTCTGCTCCTCCTGTTCGAC |
| hGAPDH/R | GCGCCCAATACGACCAAATC |
| hGAP43/F | GGAGAAGGCACCACTACTGC |
| hGAP43/R | GGCGAGTTATCAGTGGAAGC |
| hNSE | GCCCCCAATATCCTGGAGAAC |
| hNSE | TATCGGGAAGGATCAGTGGGA |

| **Primers to make DNA templates for an RNA probe P−Y123** | |
| --- | --- |
| Forward | TAATACGACTCACTATAGGGAGCCCCGCCCCTGCCGGC |
| Reverse | GTACCTTGCGGGATTTCTGGCCTCCGG |

**Supplementary Table S2. siRNA sequences**

| **siRNA** | **5’−3’ Sequence** |
| --- | --- |
| si−PTBP1 | GCCUCUUUAUUCUUUUCGG |
| si−PTBP2 | GAGAGGAUCUGACGAACUA |
| si−HNRNP H/F | GGAAGAAAUUGUUCAGUUC |
| si−YB1 | AGAAGGUCAUCGCAACGAATT |

The control siRNA was AllStar Negative Control siRNA (1027281) by Qiagen.

**Supplementary Table S3. Antibodies for immunoblotting**

| **Primary antibodies for immunoblotting** | |
| --- | --- |
| PTBP1(goat polyclonal N−20) | Cat # sc−16547, 1:1000, Santa Cruz biotechnology |
| PTBP2 (goat polyclonal T−14) | Cat # sc−103849, 1:1000, Santa Cruz biotechnology |
| U2AF65 (mouse monoclonal MC3) | Cat # sc−53942, 1:400, Santa Cruz biotechnology |
| GAPDH (rabbit polyclonal) | Cat # G9545, 1:1000, Sigma−Aldrich |
| FLAG (mouse monoclonal M2) | Cat # F1804, 1:1000, Sigma−Aldrich |
| **Secondary antibodies for immunoblotting** | |
| Goat anti−mouse IgG (HRP−linked antibody) | Cat # sc−2354, 1: 5000, Santa Cruz Biotechnology, |
| Anti−rabbit IgG (HRP−linked antibody) | Cat # 7074, 1: 5000, Cell Signaling Technology |
| Anti−mouse IgG (HRP−linked antibody) | Cat # 7076, 1:5000, Cell Signaling Technology |

**Supplementary Materials and Method** (Related to Supplementary Fig. S8)

**Isolation of recombinant proteins**

Human PTBP1 and U2AF65 cDNAs were cloned into pGEX−6P−1 vector having a N−terminal GST (glutathione S−transferase) tag, to make pGEX−6P−1−PTBP1 and pGEX−6P−1−U2AF65, respectively. *Escherichia coli* (DH5a) transformed with either vector was cultured at 37°C, and protein expression was induced by adding 0.04 mM IPTG for 4 h at 37°C. Cells were centrifuged and lysed in buffer A [50 mM Tris (pH 8.0), 200 mM NaCl, and 1 mM EDTA] containing 1 mg/ml lysozyme and 1% Triton X−100 on ice for 30 min. After centrifugation at 10,000 × *g* for 30 min, the supernatant was collected and incubated with Glutathione Sepharose 4B beads (Cytiva) at 4°C for 1 h. To release the proteins from the beads, the beads were washed with cleavage buffer [100 mM NaCl, 50 mM Tris−HCl (pH 8.0), 1 mM EDTA, and 1 mM DTT] 3 times and then incubated in cleavage buffer containing PreScission Protease (Cytiva, Global life sciences solutions operations, Buckinghamshire, UK) at 4°C for 16 h. The treated beads were centrifuged, and the supernatant was harvested.

**UV−crosslinking and competition assay**

Biotin−labeled RNA probe (P−Y123) was synthesized with the T7 RiboMAX large−scale RNA production system (Promega, Madison, Wisconsin, United States), using a genomic *AGRN* template containing Y exon and its 134−nt upstream intronic region covering P−Y1, P−Y2, and P−Y3 sites (**Supplementary Fig. S8A**). Biotin−labeled RNA probe (P−Y123) was incubated with recombinant U2AF65 with or without recombinant PTBP1 at 30°C for 15 min in a 20−μl reaction mixture containing 10 mM HEPES−KOH (pH 7.9), 100 mM KCl, 1 mM dithiothreitol, 10% glycerol, and 0.02% NP−40. After UV crosslinking (1200 J/cm^2^), the samples were treated with 0.25 U Rnase A and 10 U Rnase T1 (Ambion Rnase Cocktail) at 37°C for 30 min and were boiled in Laemmli buffer. Then, samples were separated on 7.5% SDS−PAGE, transferred to a membrane followed by blocking with 5% skimmed milk. Membranes were then incubated with the streptavidin−horseradish peroxidase conjugate (RPN1231−2ML, Cytiva, Global life sciences solutions operations, Buckinghamshire, UK) overnight and detected with Amersham ECL Western blotting detection reagents (GE Healthcare, Global life sciences solutions operations, Buckinghamshire, UK).

**Supplementary Figure**

**
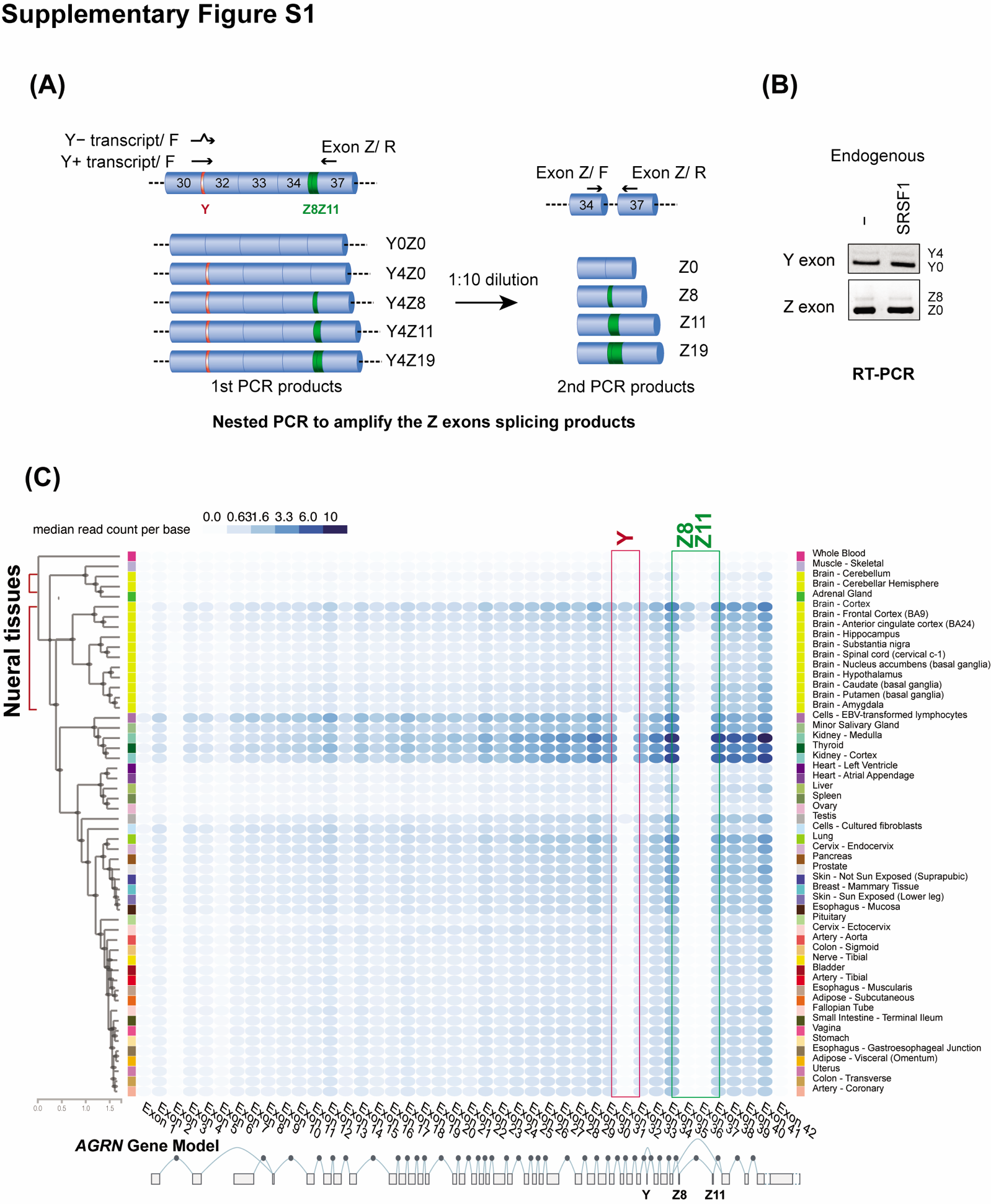
**

**Supplementary Figure S1: Splicing of Y and Z exons in human tissues**

(**A)** Schematic showing the isoform−specific nested RT−PCR. In the first round of RT−PCR, Y− and Y+ transcripts were amplified using Y− transcript/F and Y+ transcript/F primers, respectively, and a common reverse primer (Exon Z/R). Y− transcript/F lacks and Y+ transcript/F contains the 12 nt sequence of Y exon to specifically bind to the Y− and Y+ transcripts, respectively. In the second round of RT−PCR, the first RT−PCR product was diluted 10 times and was further amplified using a primer pair (Exon Z/F and Exon Z/R) spanning Z exons.

(**B)** RT−PCR showing alternative splicing of endogenous Y and Z exons in SH−SY5Y cells introduced with or without SRSF1 overexpression vector.

(**C)** Heatmap of exon expression levels of human *AGRN* in 54 human tissues by the Genotype−Tissue Expression (GTEx) project (https://www.gtexportal.org/home/gene/AGRN). Exons are indicated in the horizontal axis, and tissues are indicated in the right vertical axis. The intensity of blue color indicates the median read count per base, which represents the expression level of each exon. Y, Z8, and Z11 exons are exons 32, 36, and 37, respectively. Brain tissues are labeled as "neural tissues” on the left. Exon/intron structure of the *AGRN* gene is schematically shown at the bottom.


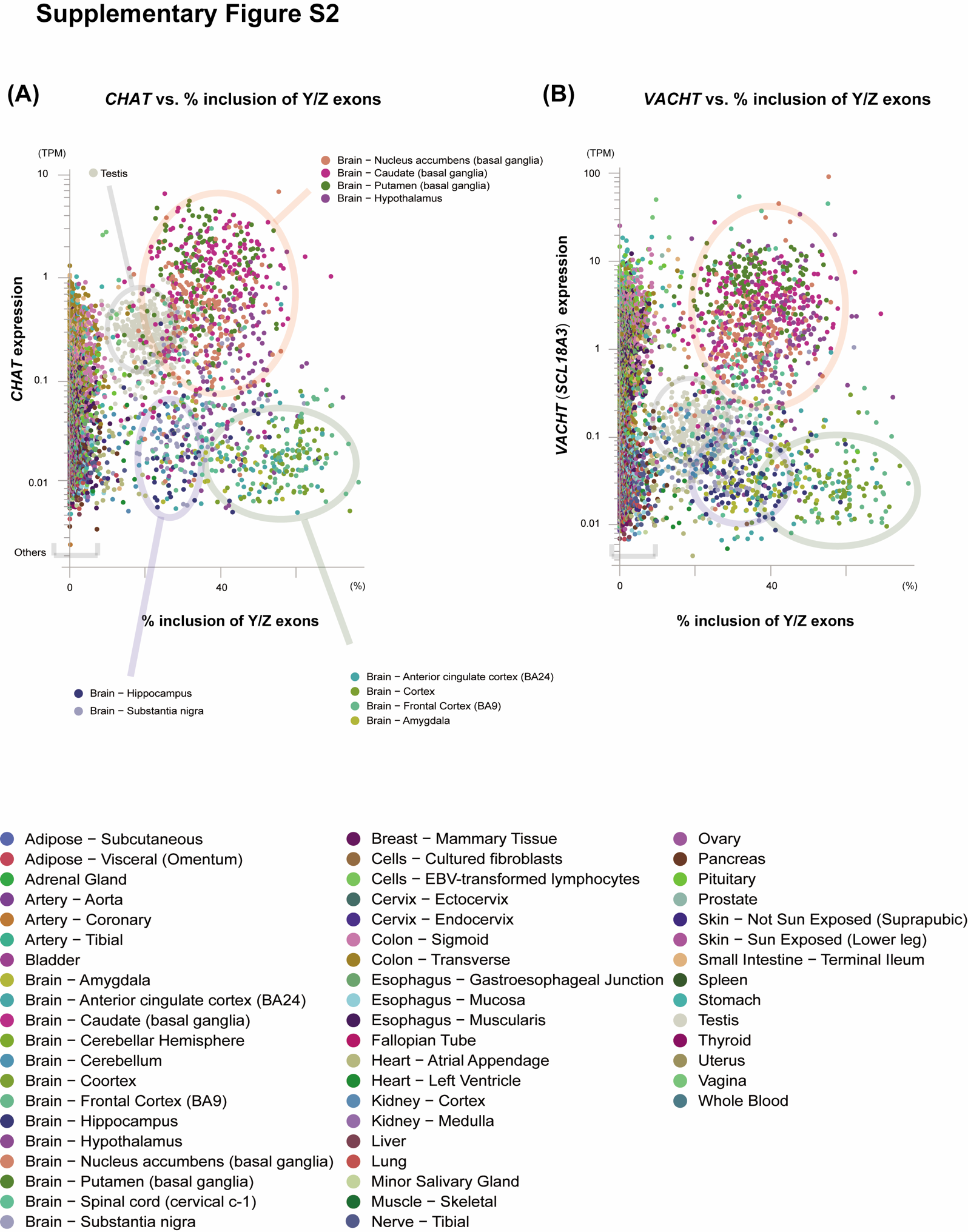


**Supplementary Figure S2: Splicing of Y and Z exons and the expression of cholinergic marker genes in human tissues**

Scatter plot showing the relationship between the inclusion level of Y and Z exons and the expression level of cholinergic markers; *CHAT* (A) and *VACHT* (B), in RNA−seq data of 54 human tissues. TPM (transcript per million) values of *CHAT* and *VACHT* (*SLC18A3*), and three representative *AGRN* isoforms; ENST00000379370.6 (Y0Z0 isoform), ENST00000620552.4 (Y4Z19 isoform) and ENST00000419249.2 (another Y4Z19 isoform with the truncated 5’ and 3’ ends) were extracted from RNA−seq data in the GTEx portal (<https://gtexportal.org/home/datasets>). Y4Z8 and Y4Z11 isoforms were not detected in the GTEx RNA−seq analysis.

%Inclusion of Y/Z exons was calculated as:

%Inclusion of Y/Z exons = [(TPM of ENST00000620552.4 + TPM of ENST00000419249.2) / (TPM of ENST00000379370.6 + TPM of ENST00000620552.4 + TPM of ENST00000419249.2)] x 100.

Each dot represents an RNA−seq data of a tissue sample, and the different colors indicate different tissues. Four noticeable clusters are indicated by open circles.


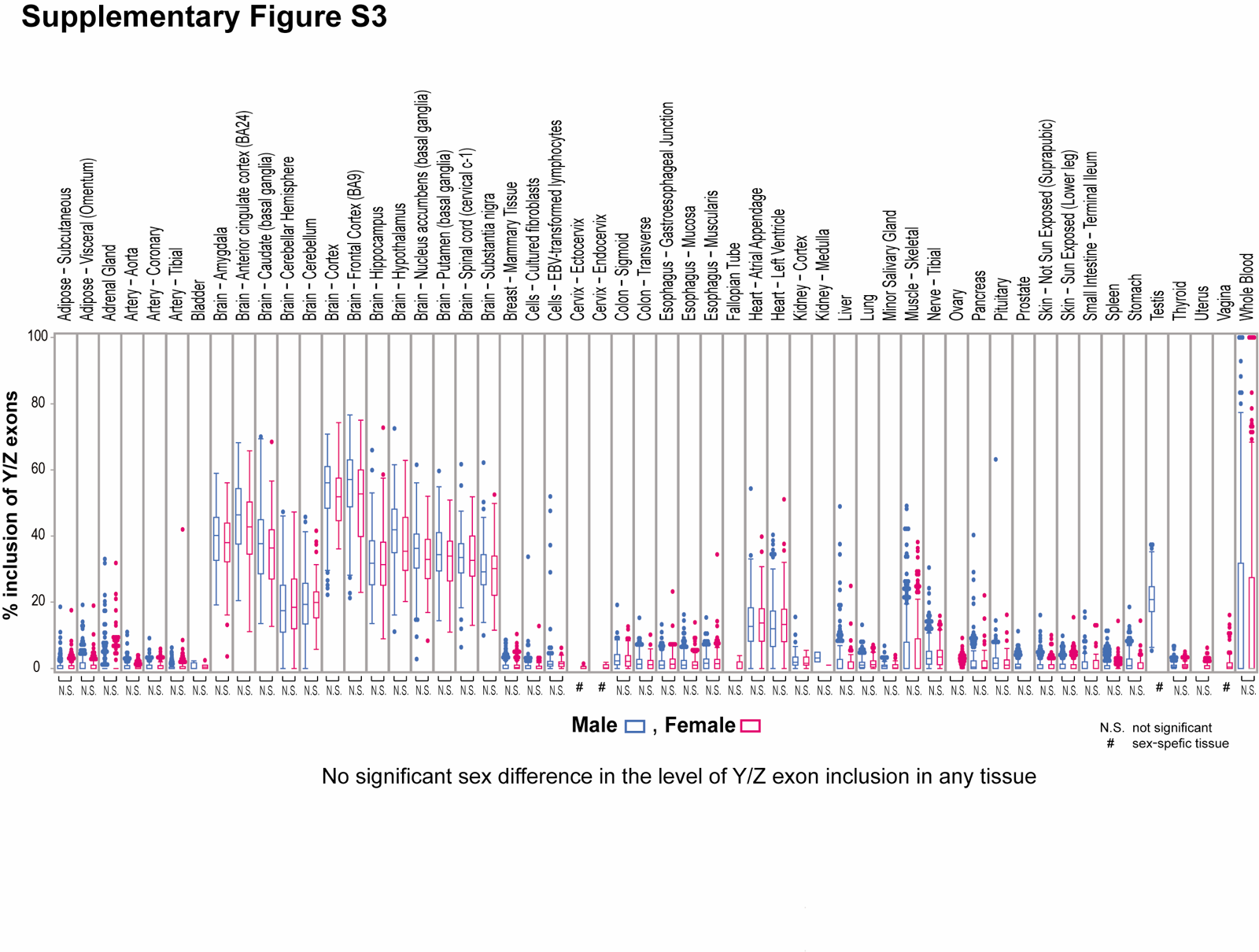


**Supplementary Figure S3: Splicing of Y and Z exons in male and female human tissue samples**

Box plot showing the inclusion level of Y and Z exons in RNA−seq data of male (blue) and female (red) samples from 54 different human tissues. TPM values of three representative *AGRN* isoforms were extracted from RNA−seq data in the GTEx portal (<https://gtexportal.org/home/datasets>) and %inclusion of Y/Z exons (vertical axis) was calculated as in **Supplementary Fig. S2**. No significant sex difference was observed in the inclusion level of Y and Z exons in any of the analyzed tissues by Wilcoxon signed rank sum test with Bonferroni correction. N.S., not significant; #, sex−specific tissue.

**
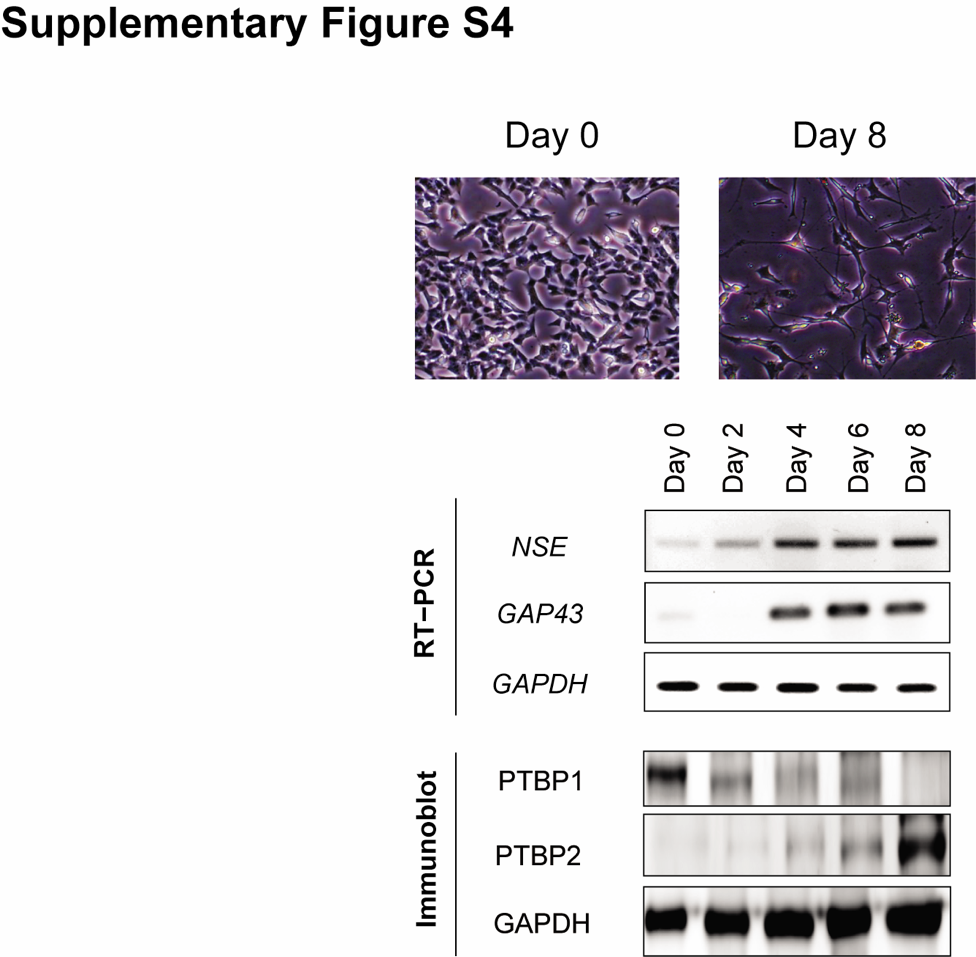
**

**Supplementary Figure S4: Downregulation of PTBP1 during neuronal differentiation**

SH−SY5Y cells are differentiated for 8 days with 10 μM retinoic acid. Representative image of undifferentiated cells at day 0 and differentiated cells at day 8. RT−PCR (upper three panels) of *GAPDH* and neuronal differentiation markers, *NSE* and *GAP43*, during differentiation of SH−SY5Y cells. Immunoblots (bottom three panels) of PTBP1, PTBP2 and GAPDH.


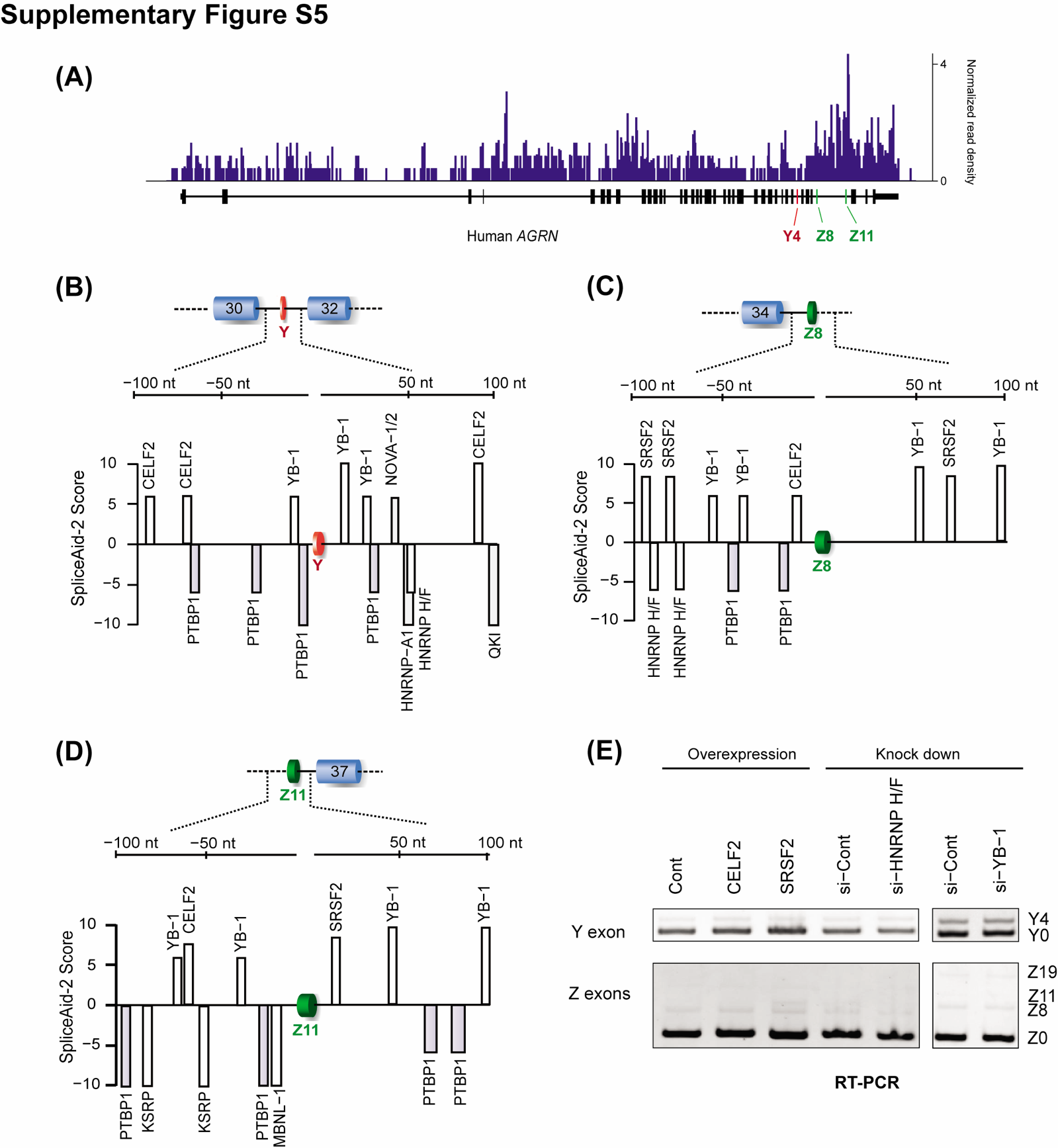


**Supplementary Figure S5: Identification of splicing *cis*−elements and their cognate RBPs by SpliceAid−2**

(**A)** Read distribution of PTBP1−eCLIP on chr1: 1,018,556−1,058,573 (GRCh38/hg38). The PTBP1−eCLIP data using K562 cells (ENCFF258TKH.bigWig) was downloaded from the ENCODE portal [28] (https://www.encodeproject.org/) and was visualized with IGV browser [29]. The exon−intron structure of *AGRN* is shown at the bottom.

(**B, C & D)** Schematic showing the predicted biding sites of RBPs in 100−nt flanking introns upstream and downstream to Y **(B)**, Z8 **(C)**, and Z11 **(D)** exons. SpliceAid−2 score in the vertical axis represents the predicted binding affinity of an indicated RBP. Only sites with the SplicAid−2 score of >5 or <−5 are shown.

(**E)** RT−PCR showing splicing of endogenous Y and Z exons in SH−SY5Y cells overexpressing CELF2 or SRSF2, and those treated with siRNAs against *HNRNPH1/HNRNPH2* and *HNRNPF* (si−HNRNP H/F) or *YBX1* (YB−1).


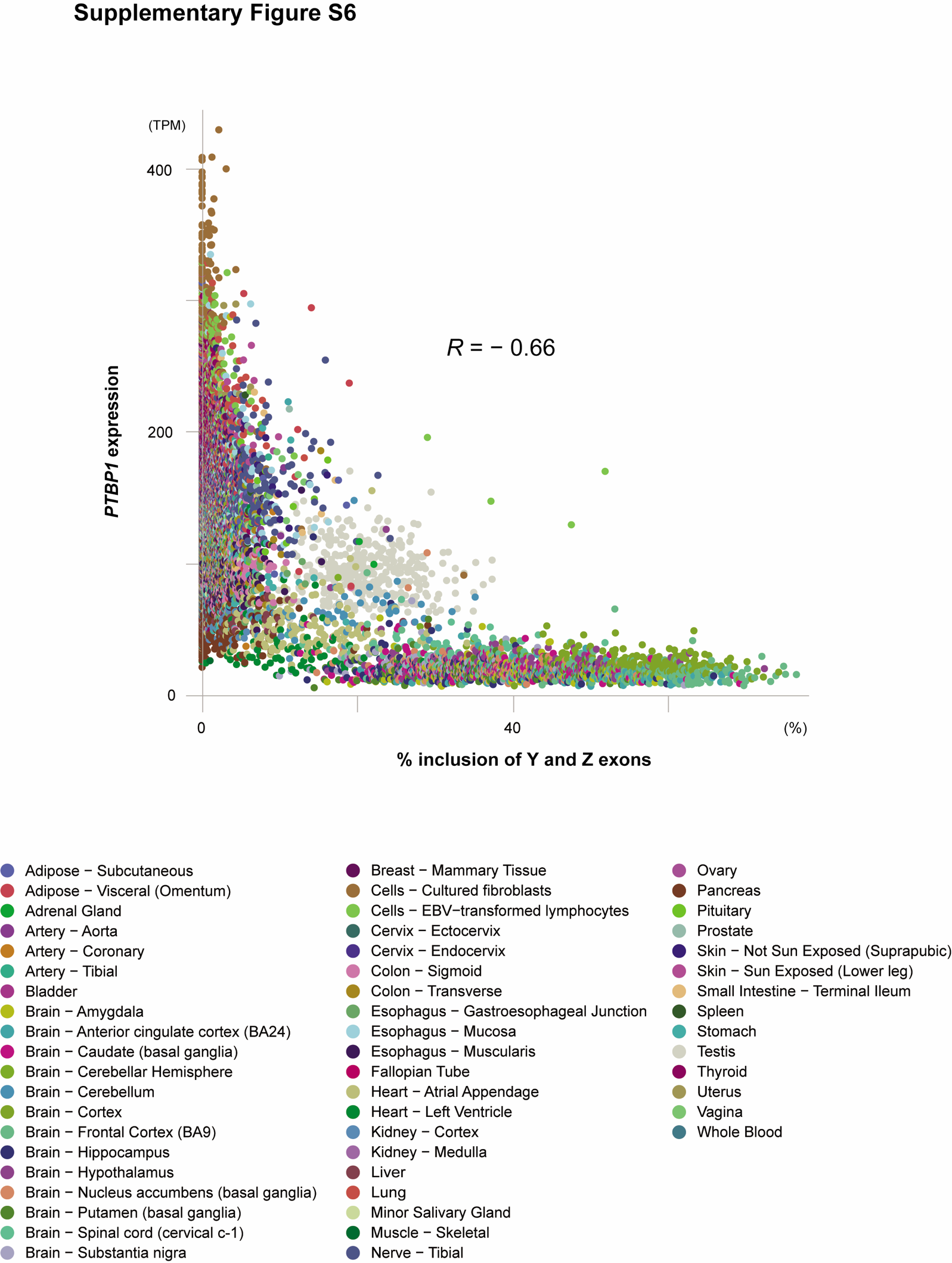


**Supplementary Figure S6:** **Splicing of Y and Z exons and *PTBP1* expression in human tissues**

Scatter plot showing the relationship between the inclusion level of Y and Z exons and the expression level of *PTBP1*, in RNA−seq data of 54 human tissues. TPM values of *PTBP1* and three representative *AGRN* isoforms were extracted from RNA−seq data in the GTEx portal (<https://gtexportal.org/home/datasets>) and %inclusion of Y/Z exons (vertical axis) was calculated as in **Supplementary Fig. S2**. Each dot represents an RNA−seq data of a tissue sample, and the different colors indicate different tissues. *R,* Pearson’s correlation coefficient.


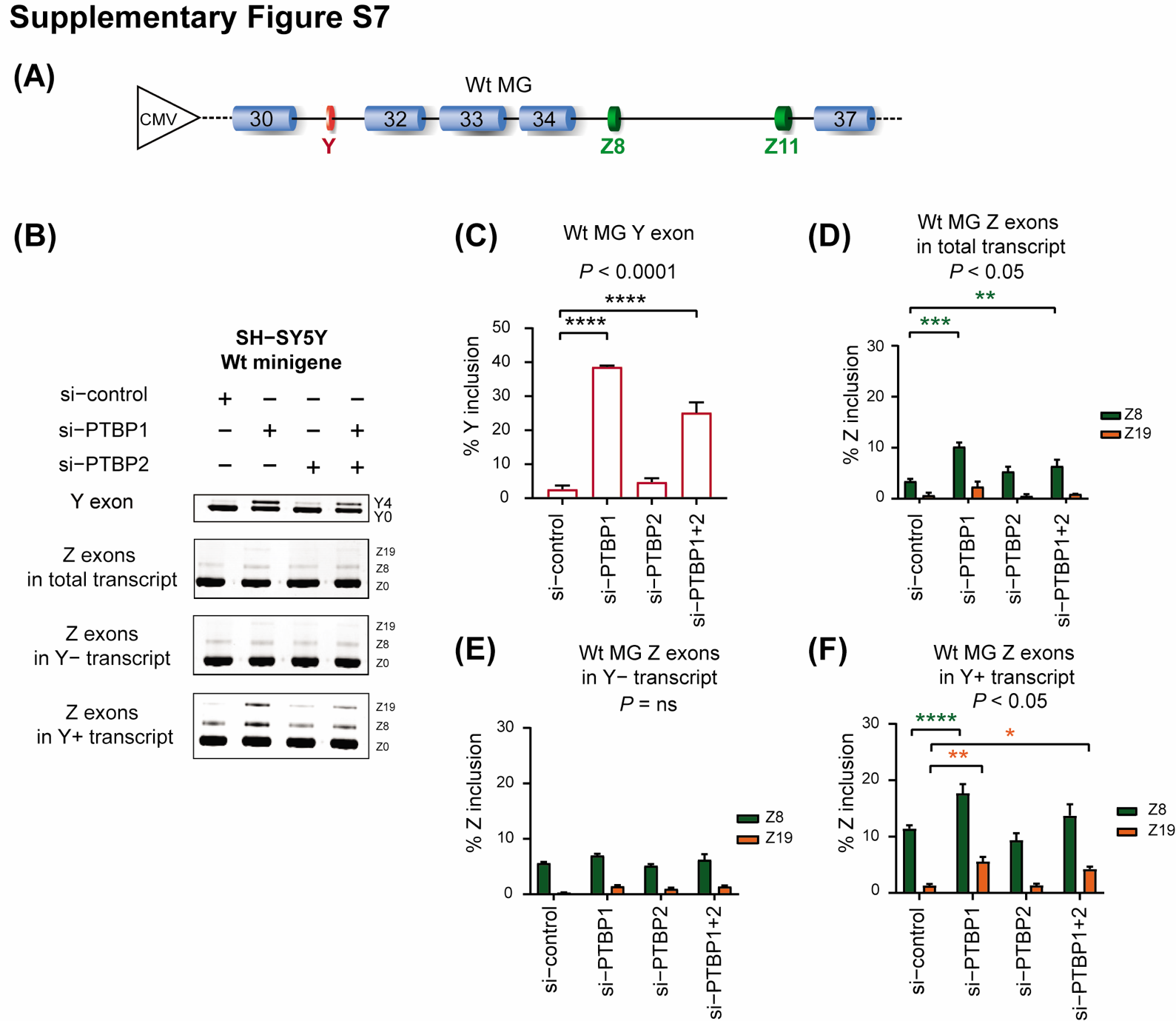


**Supplementary Figure S7: *AGRN* minigene recapitulates the endogenous splicing pattern of Y and Z exons**

(**A)** Schematic showing the human wild type *AGRN* minigene (Wt MG) spanning exons 30 to 37 introduced into pcDNA3.1+ mammalian expression vector.

(**B)** RT−PCR showing splicing of Y and Z exons of Wt MG in SH−SY5Y cells treated with indicated siRNAs.

**(C, D, E & F)** Quantification of **(B)**.

Mean and SD (*n* = 3 independent experiments) of %Y inclusion and %Z inclusion are indicated. *P*−value of one−way ANOVA **(C)** and two−way ANOVA **(D, E & F)** are indicated above the graphs. **P* <0.05, ***P* <0.01, ****P* <0.001, and *****P* <0.0001 by Tukey’s post hoc test when applicable.


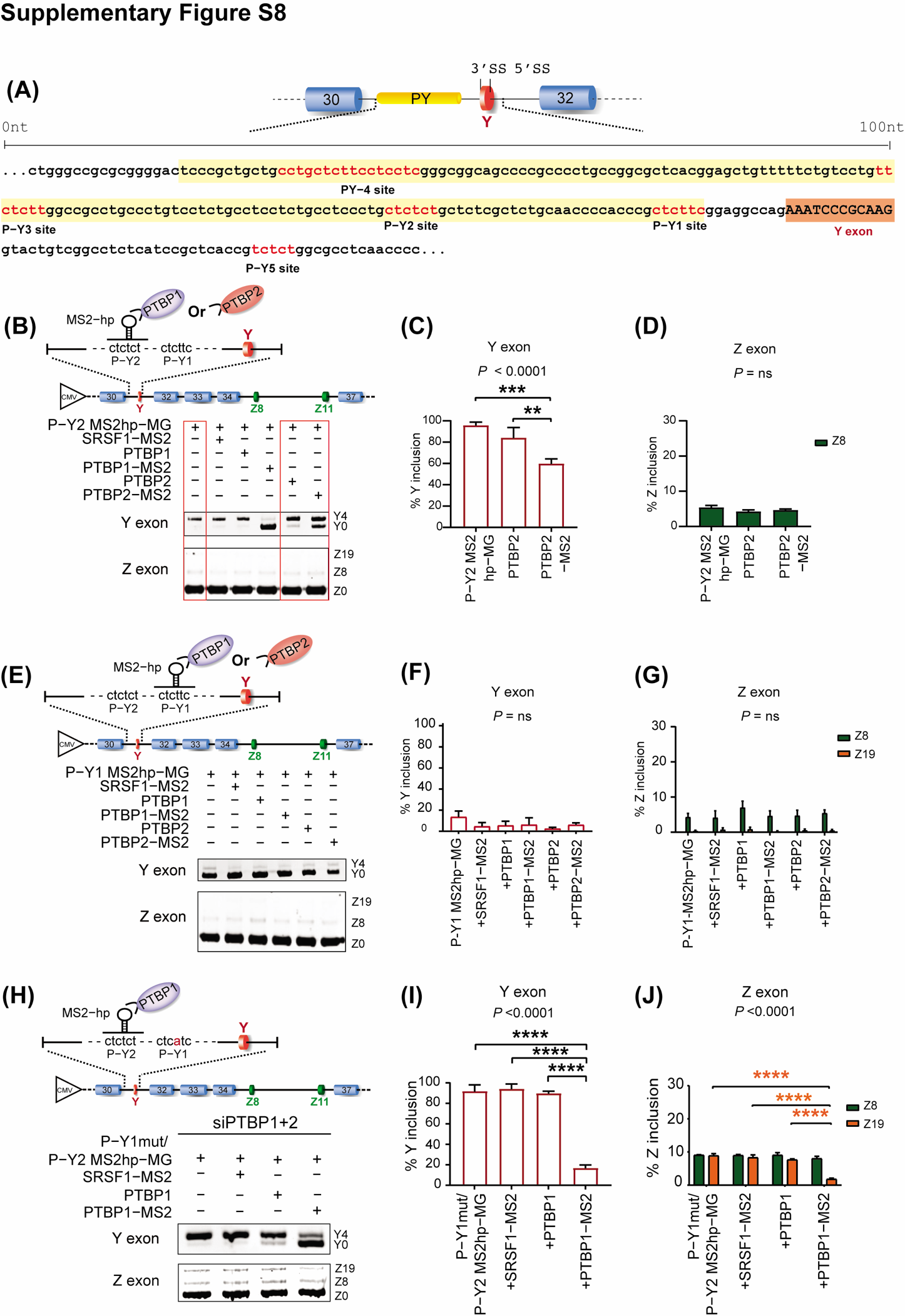


**Supplementary Figure S8: The roles of PTBP1−binding sites around Y exons for the splicing repression of Y and Z exons**

(**A)** Schematic showing Y exon and its flanking intronic regions. Y exon and its upstream polypyrimidine (PY) tract are highlighted in red and yellow, respectively. Candidate PTBP1 binding sites predicted by SpliceAid−2 are indicated in red letters.

(**B)** Schematic (top) showing artificial tethering of PTBP−MS2 coat proteins at P−Y2 in the reporter minigene, P−Y2 MS2hp−MG. Cells were co−transfected with the reporter minigene and indicated effectors. RT−PCR (bottom panel) showing splicing of Y exon and Z exons of P−Y2 MS2hp−MG in SH−SY5Y cells. Tethering of PTBP1 at P−Y2 site in lanes 1−4 is also shown in Fig 3D. In contrast, tethering of PTBP2 at P−Y2 site is shown in lanes 5−6 (red box).

(**C & D)** Quantification of **(B, lanes 1 and 5−6).**

(**E)** Schematic (top) showing the reporter minigene, P−Y1 MS2hp−MG, carrying a MS2 hairpin−loop (MS2−hp). Cells were co−transfected with the reporter minigene and indicated effectors. RT−PCR (below) showing splicing of Y and Z exons. In contrast to Fig. 3G, the P−Y2 site was intact, and *PTBP1/2* were not silenced.

**(F & G)** Quantification of **(E).**

(**H)** Schematic (top) showing the reporter minigenes P−Y1mut/ P−Y2 MS2hp−MG carrying a MS2hp at P−Y2 and mutations (red letters) at P−Y1. Cells were co−transfected with the reporter minigene and indicated effectors upon *PTBP*s knockdown. RT−PCR (below) showing splicing of Y and Z exons.

(**I & J)** Quantification of **(H).**

Mean and SD (*n* = 3 independent experiments) are indicated in the graphs. *P*−value of one−way ANOVA **(C, D, F & I**) and two−way ANOVA **(G & J)** are indicated above the graphs. ***P* <0.01, ****P* <0.001, and *****P* <0.0001 by Tukey’s post hoc test when applicable.


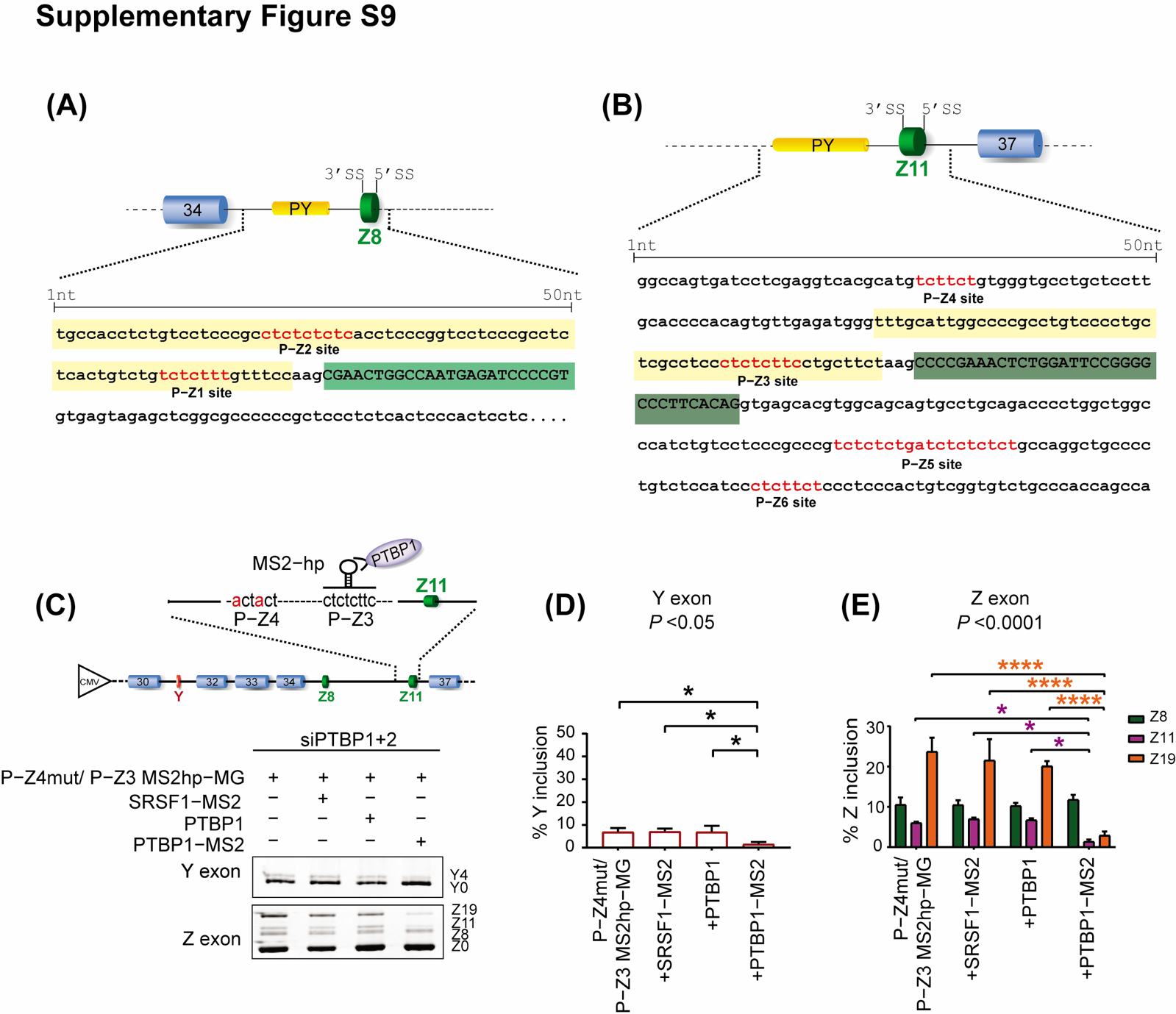


**Supplementary Figure S9: The roles of PTBP1−binding sites around Z exons for the splicing repression of Y and Z exons**

(**A & B)** Schematic showing Z8 **(A)** and Z11 **(B)** exon and their flanking intronic regions. Z8 exon, Z11 exon, and their upstream polypyrimidine (PY) tracts are highlighted in light green, dark green, and yellow, respectively. Candidate PTBP1 binding sites predicted by SpliceAid−2 are indicated in red letters.

(**C)** Schematic (top) showing the reporter minigene, P−Z4mut/P−Z3 MS2hp−MG, carrying a MS2 hairpin−loop (MS2−hp) at P−Z3 and mutations (red letters) at the P−Z4 site. Cells were co−transfected with the reporter minigene and indicated effectors upon *PTBPs* knockdown. RT−PCR (below) showing splicing of Y and Z exons.

(**D & E)** Quantification of **(C)**. Mean and SD (*n* = 3 independent experiments) are indicated in the graphs. *P*−value of one−way ANOVA **(D**) and two−way ANOVA **(E)** are indicated above the graphs. **P* <0.05 and *****P* <0.0001 by Tukey’s post hoc test when applicable.

**
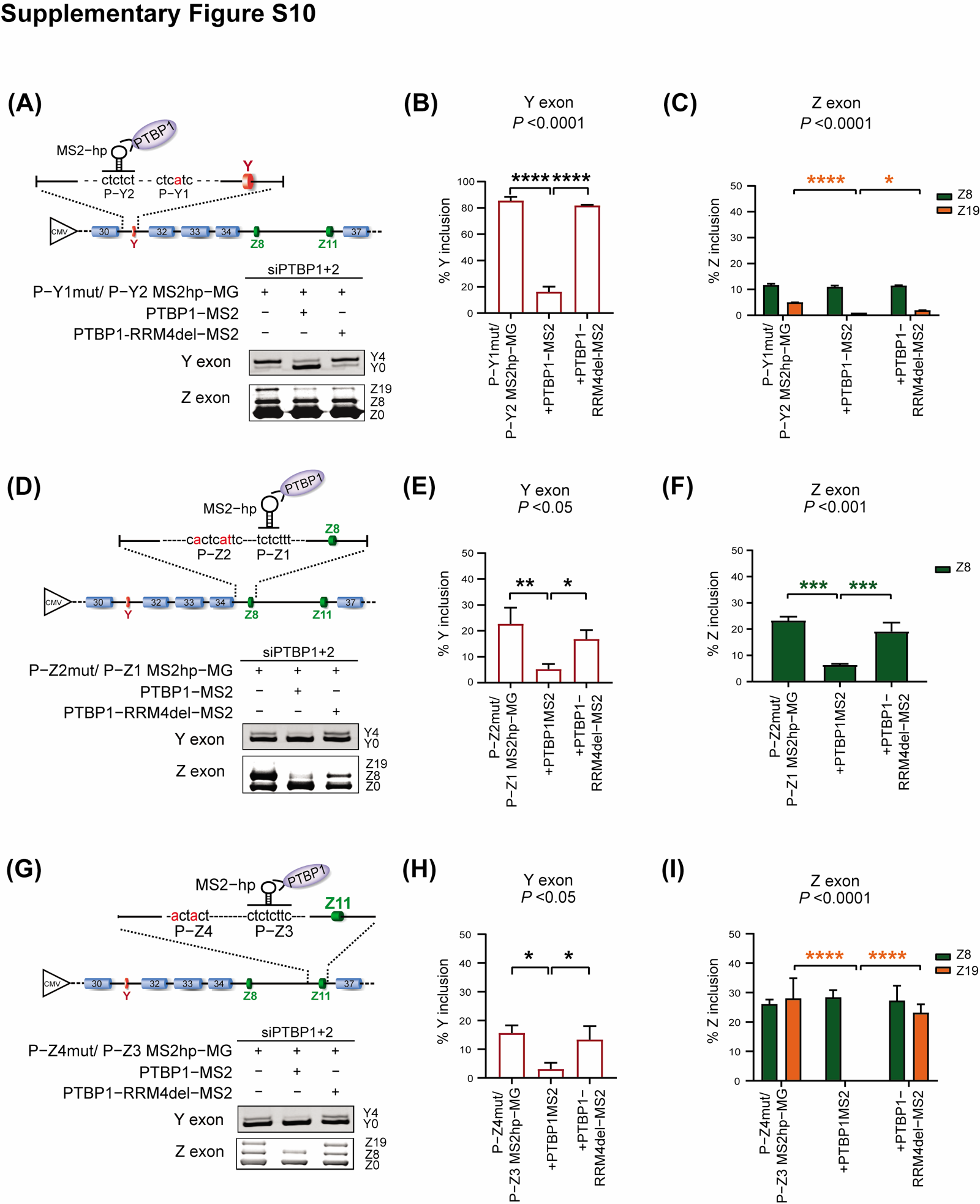
**

**Supplementary Figure S10: The essential role of RRM4 domain in PTBP1 for the repression of both Y and Z exons**

(**A, D & G)** PTBP1−MS2 and PTBP1−RRM4del−MS2 are tethered at P−Y2 in P−Y1mut/P−Y2 MS2hp−MG **(A)**, P−Z2mut/P−Z1 MS2hp−MG **(D)**, and P−Z4mut/P−Z3 MS2hp−MG **(G)** upon *PTBP*s knockdown. RT−PCR (below) showing splicing of Y and Z exons.

(**BC, EF & HI)** Quantification of **(A, D & G)**, respectively.

Mean and SD (*n* = 3 independent experiments) of %Y **(B, E & H)** and %Z **(C, F & I)** inclusion are indicated. *P*−values of one−way ANOVA (**B, E, F & H**) and two−way ANOVA **(C & I)** are indicated above the graph. **P* <0.05, ***P* <0.01, ****P* <0.001, and *****P* <0.0001 by Tukey’s post hoc test when applicable.

**
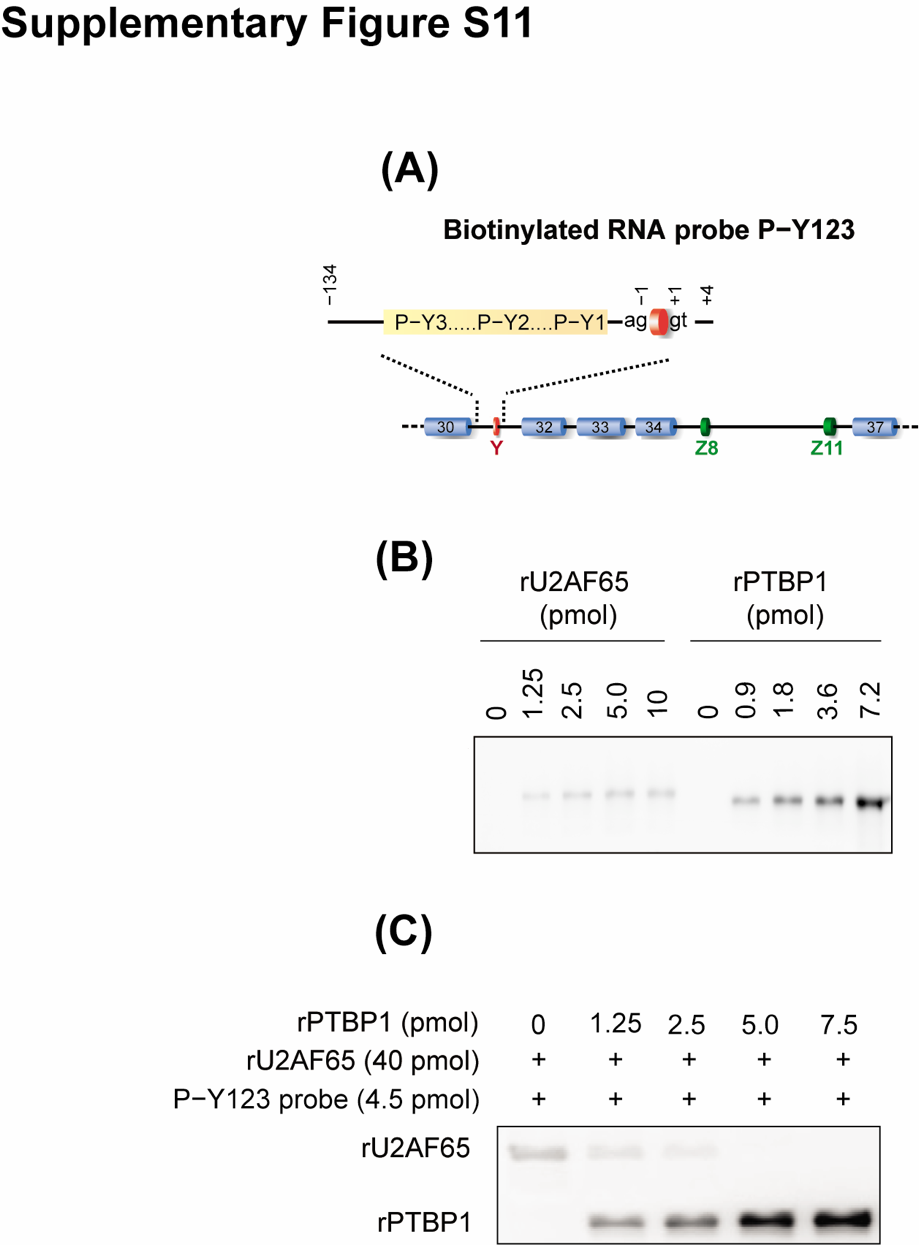
**

**Supplementary Figure S11: *In vitro* binding assay showing competition between recombinant PTBP1 (rPTBP1) and recombinant U2AF65 (rU2AF65) on P−Y123 probe**

**A)** Schematic showing sequence of the P−Y123 probe which contains Y exon and its upstream intronic polypyrimidine−rich region harboring P−Y1, P−Y2, and P−Y3 sites.

**B)** P−Y123 probe (4.5 pmol) was incubated with indicated amounts of recombinant U2AF65 (rU2AF65) or recombinant PTBP1 (rPTBP1), followed by UV−crosslinking and RNase treatment. The crosslinked proteins were separated on SDS−PAGE, transferred to a membrane, and detected by streptavidin−horseradish peroxidase conjugate. Incubation of each protein with the RNA probe showed that both rPTBP1 and rU2AF65 bound to the probe in a dose−dependent manner.

**C)** P−Y123 probe (4.5 pmol) was incubated with 40 pmol of rU2AF65 and indicated amounts of rPTBP1 for analyzing the competition between them. Crosslinked proteins were detected as **(B).** Incubation of the probe with a fixed amount of rU2AF65 and increasing amounts of rPTBP1 revealed that rPTBP1 inhibited the binding of rU2AF65 to the RNA probe in a dose−dependent manner to Y exon, and thereby suppresses its splicing.
