# Supplementary figures and images for: Neural Isoforms of Agrin Are Generated by Reduced PTBP1−RNA Interaction Network Spanning the Neuron−Specific Splicing Regions in AGRN
Source: Int J Mol Sci. 2023 Apr 18;24(8):7420. doi: 10.3390/ijms24087420 (PMC10139058; doi:10.3390/ijms24087420)

Supplementary Figure S1

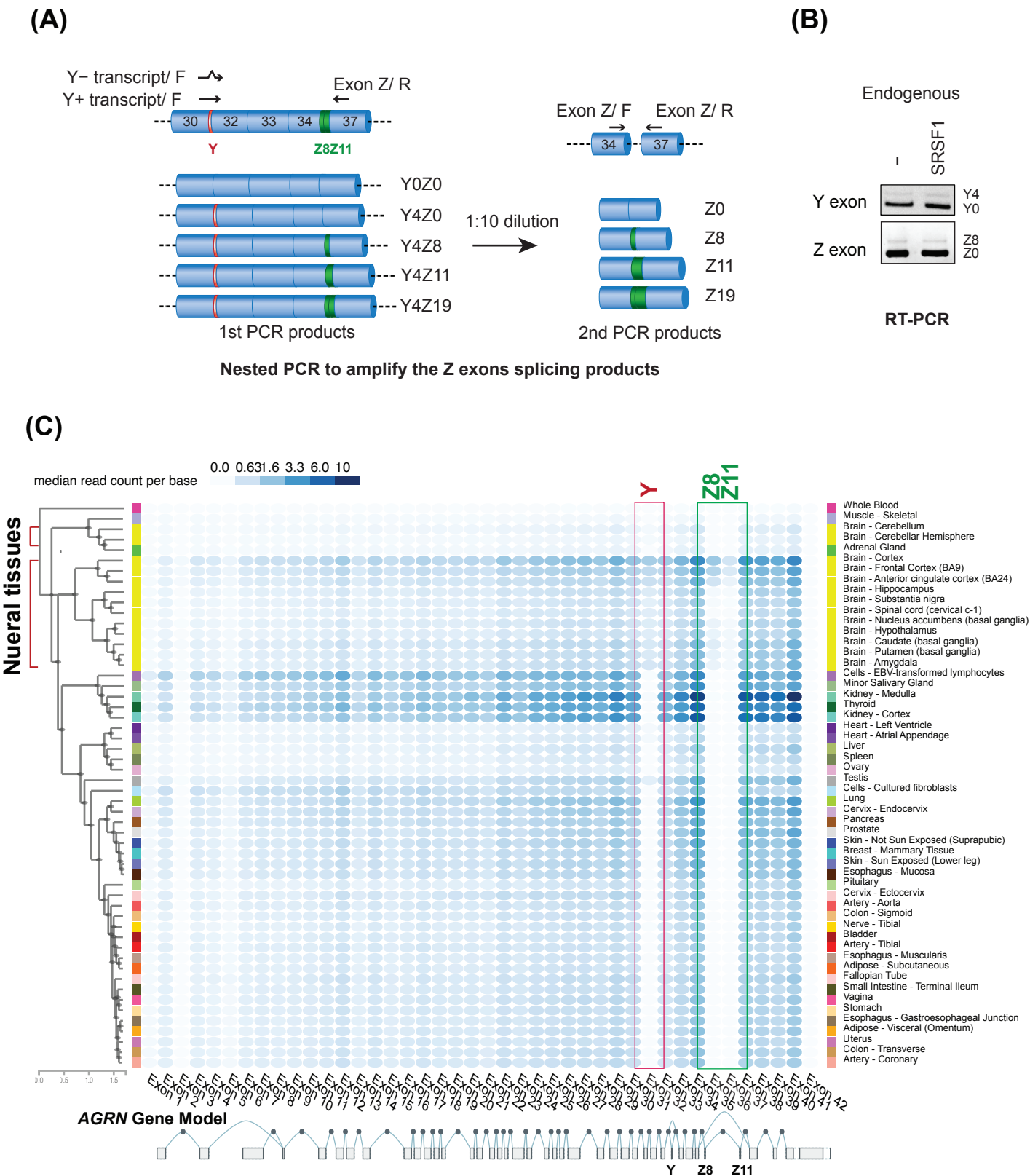

Supplement: Supplementary file 1 [file ijms-24-07420-s001.zip › ijms-2295770-supplementary/Supplementary files/Suppementary figures pdf/Supplementary figure 1.pdf]

Supplementary Figure S11

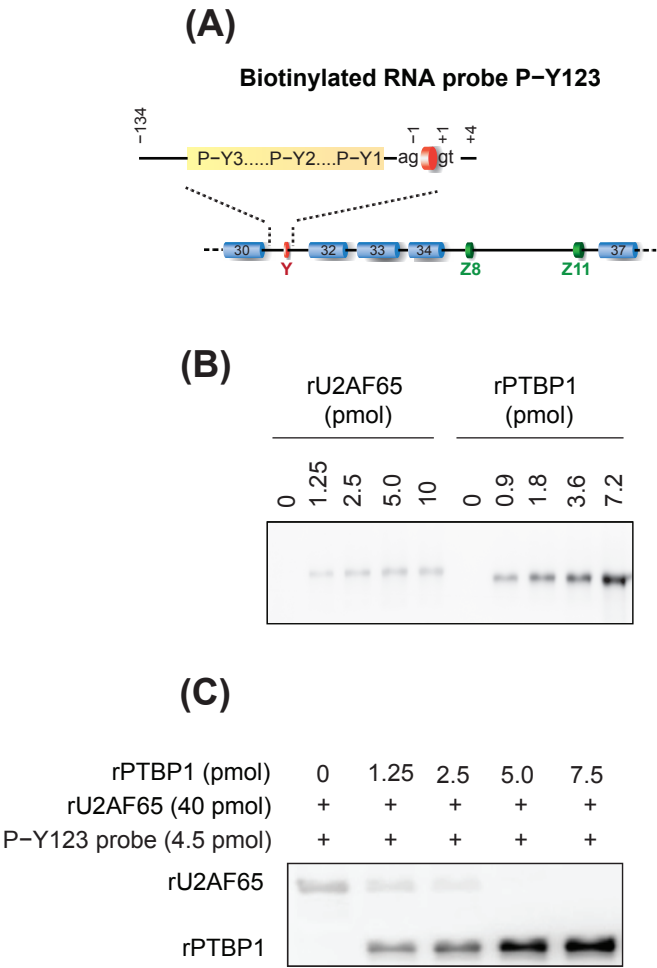

Supplement: Supplementary file 1 [file ijms-24-07420-s001.zip › ijms-2295770-supplementary/Supplementary files/Suppementary figures pdf/Supplementary figure 11.pdf]

Supplementary Figure S4

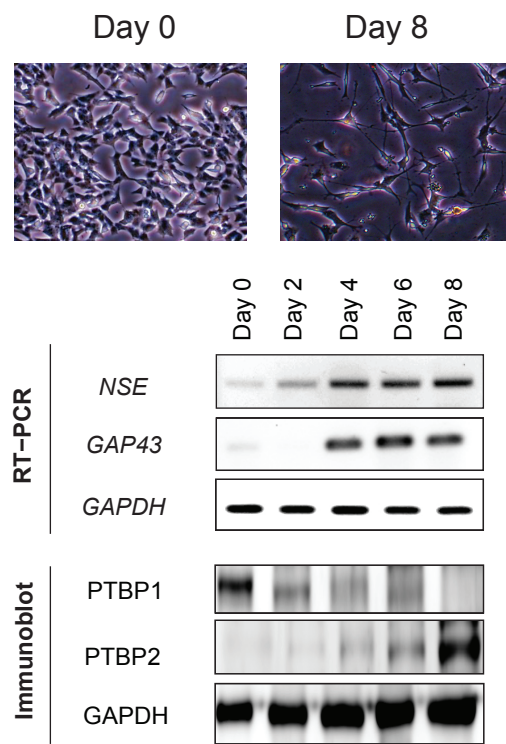

Supplement: Supplementary file 1 [file ijms-24-07420-s001.zip › ijms-2295770-supplementary/Supplementary files/Suppementary figures pdf/Supplementary figure 4.pdf]

Supplementary Figure S5

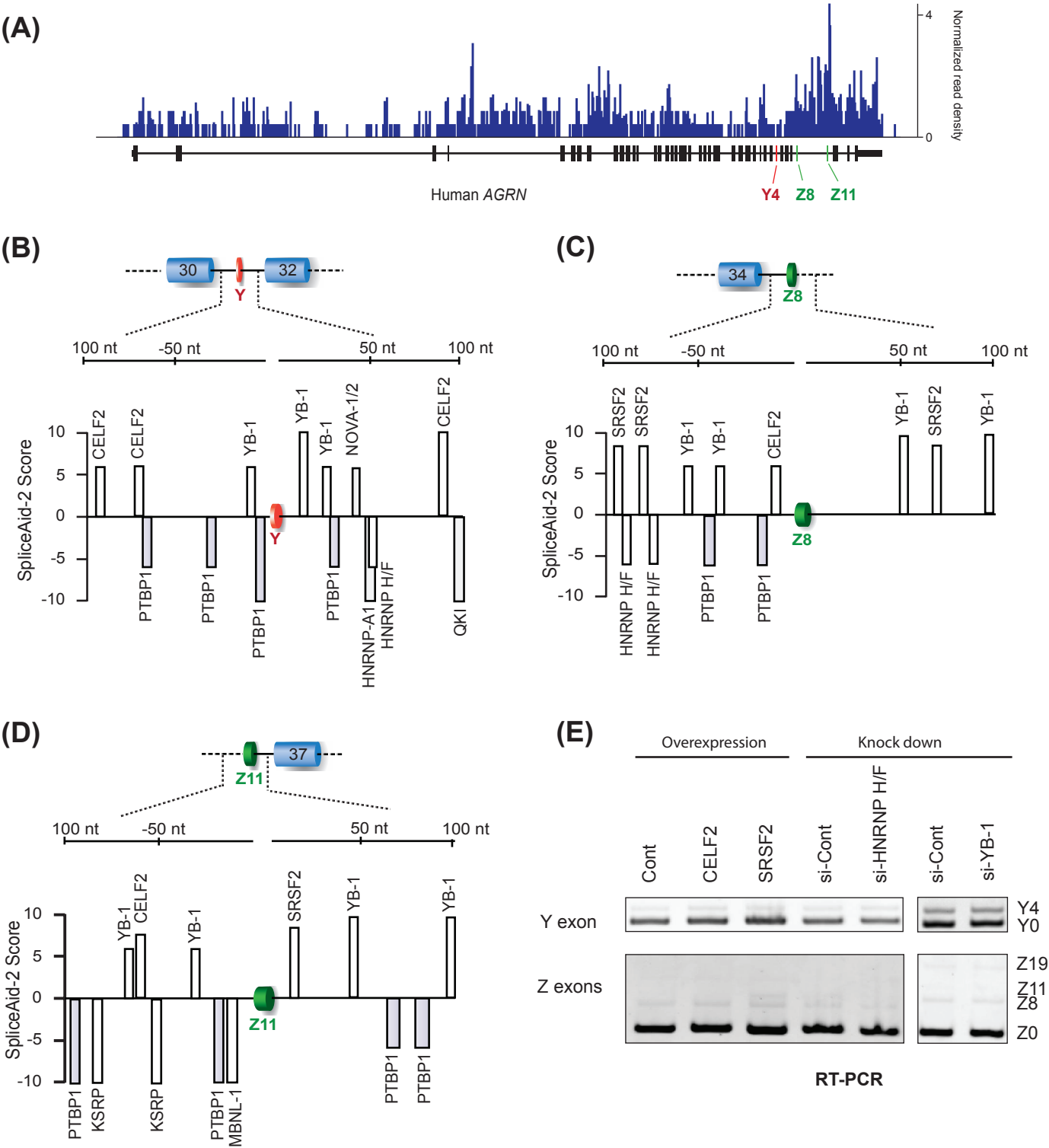

Supplement: Supplementary file 1 [file ijms-24-07420-s001.zip › ijms-2295770-supplementary/Supplementary files/Suppementary figures pdf/Supplementary figure 5.pdf]

Supplementary Figure S7

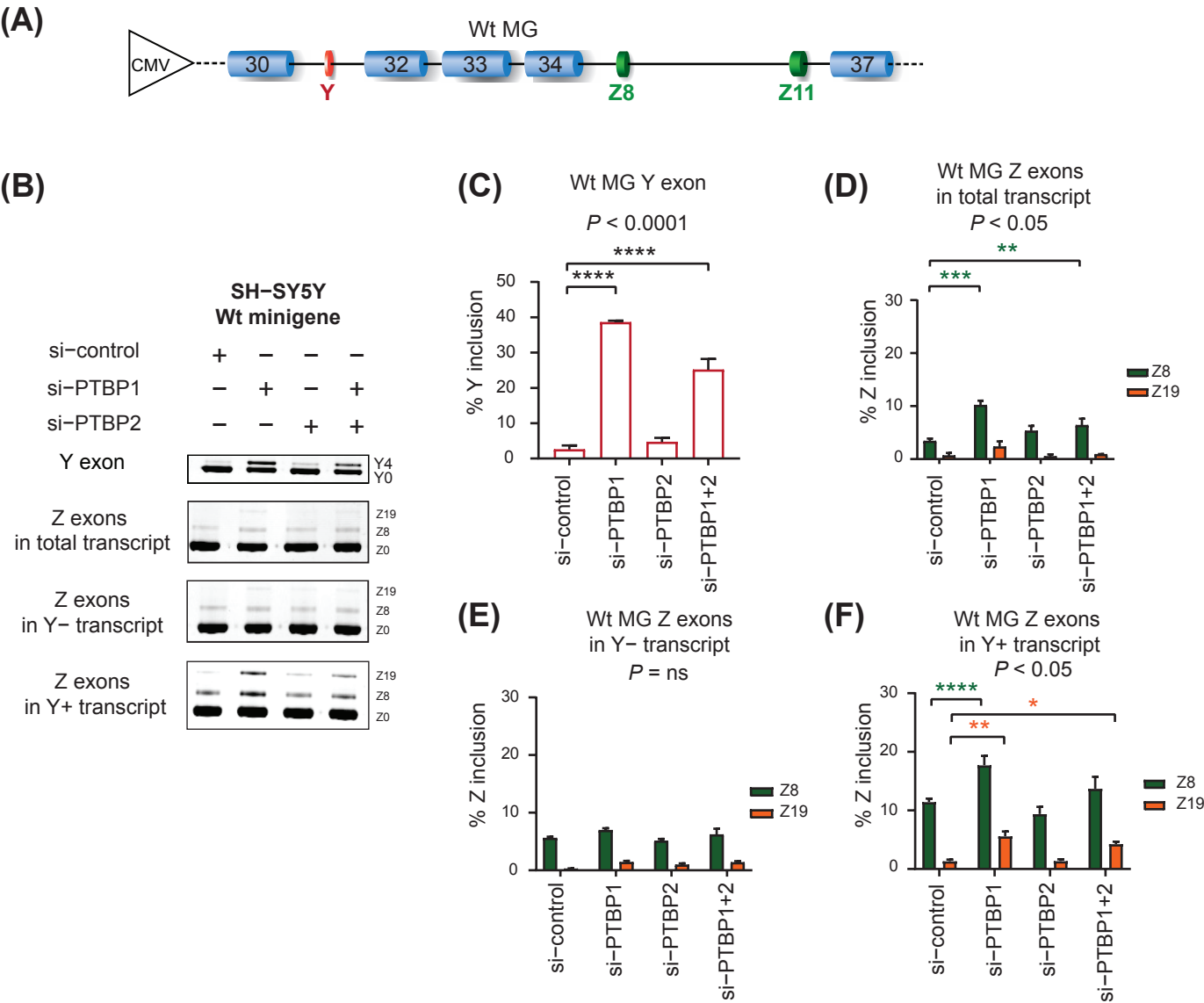

Supplement: Supplementary file 1 [file ijms-24-07420-s001.zip › ijms-2295770-supplementary/Supplementary files/Suppementary figures pdf/Supplementary figure 7.pdf]

## Supplementary Figure S8

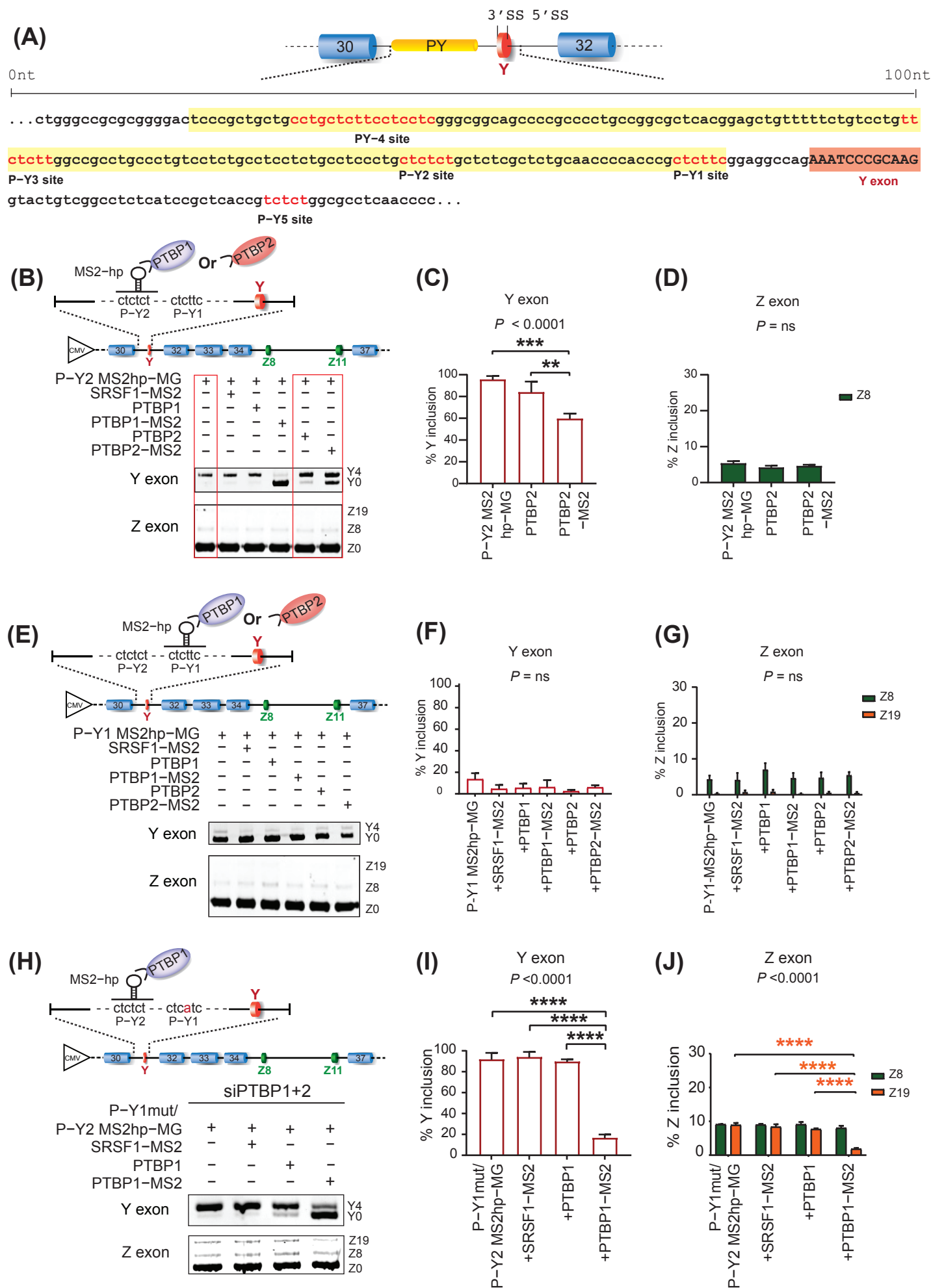

Supplement: Supplementary file 1 [file ijms-24-07420-s001.zip › ijms-2295770-supplementary/Supplementary files/Suppementary figures pdf/Supplementary figure 8.pdf]

Supplementary Figure S9

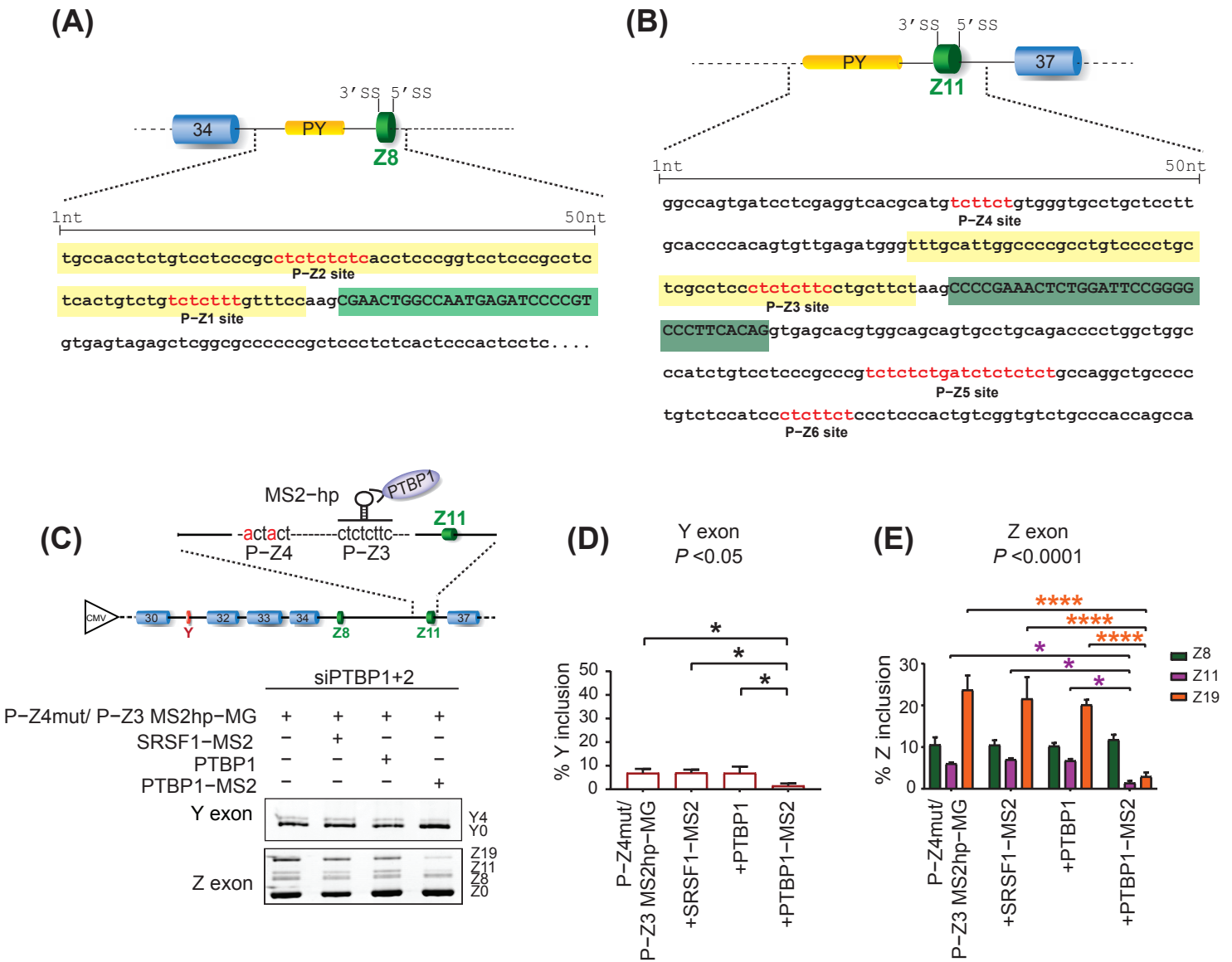

Supplement: Supplementary file 1 [file ijms-24-07420-s001.zip › ijms-2295770-supplementary/Supplementary files/Suppementary figures pdf/Supplementary figure 9.pdf]
